# Quantum Error Correction Protects Quantum Search Algorithms Against Decoherence

**DOI:** 10.1038/srep38095

**Published:** 2016-12-07

**Authors:** Panagiotis Botsinis, Zunaira Babar, Dimitrios Alanis, Daryus Chandra, Hung Nguyen, Soon Xin Ng, Lajos Hanzo

**Affiliations:** 1 Southampton Wireless, School of Electronics and Computer Science, University of Southampton, United Kingdom

## Abstract

When quantum computing becomes a wide-spread commercial reality, Quantum Search Algorithms (QSA) and especially Grover’s QSA will inevitably be one of their main applications, constituting their cornerstone. Most of the literature assumes that the quantum circuits are free from decoherence. Practically, decoherence will remain unavoidable as is the Gaussian noise of classic circuits imposed by the Brownian motion of electrons, hence it may have to be mitigated. In this contribution, we investigate the effect of quantum noise on the performance of QSAs, in terms of their success probability as a function of the database size to be searched, when decoherence is modelled by depolarizing channels’ deleterious effects imposed on the quantum gates. Moreover, we employ quantum error correction codes for limiting the effects of quantum noise and for correcting quantum flips. More specifically, we demonstrate that, when we search for a single solution in a database having 4096 entries using Grover’s QSA at an aggressive depolarizing probability of 10^−3^, the success probability of the search is 0.22 when no quantum coding is used, which is improved to 0.96 when Steane’s quantum error correction code is employed. Finally, apart from Steane’s code, the employment of Quantum Bose-Chaudhuri-Hocquenghem (QBCH) codes is also considered.

Moore’s law is expected to delve in the quantum world in the early 2020 s, since quantum effects will appear when trying to further shrink the scale of the integration in classic chips^1^. Quantum computing may be one of the ways forward, promising substantial speed-up in some applications, when compared to the existing classical solutions. Grover’s Quantum Search Algorithm (QSA)^2^,^3^ succeeds in finding a specific desired entry out of the *N* entries in an unsorted database with ~100% success probability, after evaluating as few as 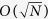 entries. One of the main assumptions that are adopted for achieving this near-perfect success is that the quantum bits or *qubits*, which take part in Grover’s QSA, will only have their quantum state changed, if they pass through quantum gates, as described in the postulates of quantum mechanics^4^,^5^,^6^. In other words, in order to achieve ~100% success probability, the qubits experience no bit- or phase-flips between gates.

Quantum computing and quantum search algorithms may be beneficially exploited in diverse large-scale applications in wireless communications, such as multiple stream detection^7^,^8^,^9^,^10^,^11^ or routing^12^,^13^. Numerous challenging optimization problems in wireless communications will be solved more efficiently by quantum search algorithms relying on Grover’s QSA. In the Supplementary Section 3, we state a number of applications that may benefit from the employment of quantum search algorithms. However, when quantum computing systems become a wide-spread commercial reality, it is expected that errors will occur in the quantum circuits, due to the inevitable presence of quantum noise, conventionally termed as *decoherence*^4^. More precisely, decoherence is due to the deleterious interaction of the constituent qubits with the environment, which perturbs the flawless superposition of states^14^,^15^,^16^,^17^,^18^. The resultant errors, occurring between the application of two quantum gates even when highly fault-tolerant gates^16^,^17^,^18^ are employed, may be modelled by the so-called depolarizing channels.

Similarly to classical error correction codes, the errors in the quantum domain may be corrected by employing quantum error correction codes. More explicitly, up to a limit, quantum codes rectify the impact of quantum noise for the sake of ensuring that the qubits retain their coherent quantum state with a high fidelity, which is a measure of “closeness” of two quantum states^19^, thus in effect beneficially increasing the coherence time of the unperturbed quantum state. This has been experimentally demonstrated in refs 20, 21, 22. The inception of quantum codes dates back to 1995 when Shor^14^ conceived the first quantum code, which was however only capable of correcting a single error. Since then the quest for approaching the quantum capacity bounds has continued. In this context, the astounding performance of quantum turbo codes^23^,^24^,^25^, quantum-domain low density parity check codes^26^,^27^,^28^ and quantum polar codes^29^,^30^, which rely on long streams of information qubits, is of particular significance. However, we will argue that from an implementation-oriented perspective and particularly for application in Grover’s QSA, the employment of short block codes is more feasible. Hence, in this treatise, we invoke Steane’s Code^31^ and Quantum Bose-Chaudhuri-Hocquenghem (BCH) codes^32^,^33^,^34^ for improving the search-success probability of Grover’s QSA, by substantially reducing the qubit error ratio of each link between a pair of serially concatenated gates.

Various noise models impairing Grover’s QSA have been proposed^35^,^36^,^37^,^38^,^39^,^40^,^41^ and some also have employed quantum error correction^35^,^42^. However, there is no study on the effect of depolarizing channels in diverse locations of the index register in generic Grover architectures, with no prior knowledge of the solution index, in conjunction with Steane’s code or the QBCH code employed and evaluated in multiple search scenarios. Based on the aforementioned background, our novel contributions are:

1. We propose a system model, where depolarizing channels represent the deleterious effects of quantum errors between the employment of two consecutive quantum gates. The model is capable of describing the errors occurring to the index register in every part of Grover’s operator, while distinguishing the index and the value registers in the Oracle and without prior knowledge of the problem’s solution state.
2. We propose quantum error correction codes, such as the Steane Code^31^ and Quantum Bose-Chaudhuri-Hocquenghem (QBCH) code^32^,^33^,^34^ for detecting and correcting the qubit errors. The performance of the codes is presented in terms of Grover’s QSA’s success probability.
3. We characterize the effect that qubit errors have on the success probability of Grover’s QSA, as well as the specific effect of the location of these errors in the quantum circuit have on the success probability of Grover’s QSA. We statistically characterize the performance of Grover’s realistic imperfect QSA, in terms of the number of Grover iterations *L*, the size of the database *N* and the depolarizing probabilities.

The structure of the paper is as follows. In the following section, we analyse Grover’s idealized perfect QSA and its performance, when quantum decoherence and quantum noise is not an issue. Moreover, we investigate the effect that quantum noise has on the success probability of Grover’s QSA. Then, we propose the employment of short quantum error correction codes for combating the performance degradations. Finally, our conclusions are offered in the corresponding Section.

## Grover’s Idealized Quantum Search Algorithm

In quantum computing, the unit of quantum information is the qubit |*q*〉, which may be found in the quantum states |0〉, |1〉, or a superposition of |0〉 and |1〉 as in |*q*〉 = *a*|0〉 + *b*|1〉, where the amplitudes of the quantum states *a* and *b* satisfy |*a*|^2^ + |*b*|^2^ = 1 and 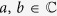. When a *measurement* or *observation* of a qubit |*q*〉 takes place, it may be found in the state |0〉 with |*a*|^2^ probability or |1〉 with |*b*|^2^ probability. The evolution of quantum states is manipulated with the aid of unitary operators or gates^4^. One of the most commonly used unitary gates is the Hadamard operator *H*, which carries out the operation of 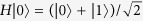 and 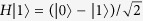. Multiple qubits may be processed together for forming composite systems. For example, two qubits |*q*_1_*q*_2_〉 may be found in the general state |*q*_1_*q*_2_〉 = *a*_00_|00〉 + *a*_01_|01〉 + *a*_10_|10〉 + *a*_11_|11〉, in conjunction with |*a*_00_|^2^ + |*a*_01_|^2^ + |*a*_10_|^2^ + |*a*_11_|^2^ = 1. Depending on whether the qubits of a composite system may be described separately or not, the quantum state is termed as *separable* or *entangled*^4^. For example, the 2-qubit quantum state 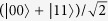 represents an entangled state, where a potential measurement of the first qubit directly determines the quantum state of the entangled qubit.

In Grover’s QSA, the amplitudes of the quantum states are real-valued, as in 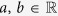. Grover’s QSA efficiently solves a search problem, where given a known value *δ*, the goal is to find a specific index *x* or address of a database of size *N*, which stores the known value *δ*. This can also be described with the aid of the function *f(x*) = *δ*. More explicitly, Grover’s QSA succeeds in finding the desired index *x* with ~100% probability of success, by observing the final composite system after applying Grover’s operator 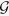, which is constituted by an *L*-fold serial concatenation of quantum gates. In order for Grover’s operator to succeed in finding the address containing the value *δ*, the number of solutions *S*, which determines the number of different database indices *x*_*i*_ that correspond to *f(x*_*i*_) = *δ*, has to be known *a priori*.

**Figure 1:**
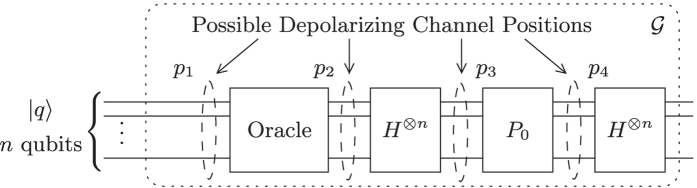
Quantum circuit of Grover’s operator 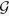, along with the potential depolarizing channels’ positions. The input quantum state |*q*〉 may either be in an equiprobable superposition of states, or the output of the previous application of Grover’s operator. The depolarizing probability of the channel in the *i*th position is equal to *p*_*i*_. The number of information qubits involved in each search is equal to 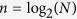, where *N* is the size of the database. An auxiliary qubit is employed in the Oracle operator.

The quantum circuit of Grover’s operator 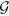
2,3 is given in Fig. 1. In the same figure, we have included the potential positions, where it will be assumed in this paper that depolarization takes place in future practical implementations of Grover’s QSA. Grover’s QSA employs 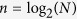 qubits and initializes them in an equiprobable superposition of all legitimate states as in


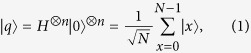


where |*x*〉 ∈ {|0〉, |1〉, …, |*N* − 1〉} is the decimal representation of the quantum states that the qubits are found in. For instance, we have |00000〉 ≡ |0〉 and |10110〉 ≡ |22〉. Initially, Grover’s operator 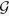 applies the Oracle *O*, which is a unitary operator that “marks” the *S* specific quantum states, which represent solutions to the search problem, by flipping their sign. In other words, the Oracle maps |*x*〉 → −|*x*〉, only if *f(x*) = *δ*. The quantum states that are not solutions of the search problem remain unaltered by the Oracle’s operation. The next three quantum gates *HP*_0_*H* of Fig. 1 describe the *diffusion* operator, which consists of the Hadamard operator applied twice and a phase flip gate, which maps |*x*〉 → −|*x*〉, only if |*x*〉 ≠ |0〉, while applying the identity operation to the quantum state |0〉. The aim of Grover’s QSA is to stop applying Grover’s operator 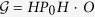 after a specific number of times *L*_*opt*_, so that the resultant probability of success is as close to unity as possible (see Supplementary Section 4). The optimal number *L*_*opt*_ of Grover iterations, which maximizes the probability of success is equal to^43^


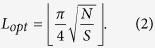


**Figure 2:**
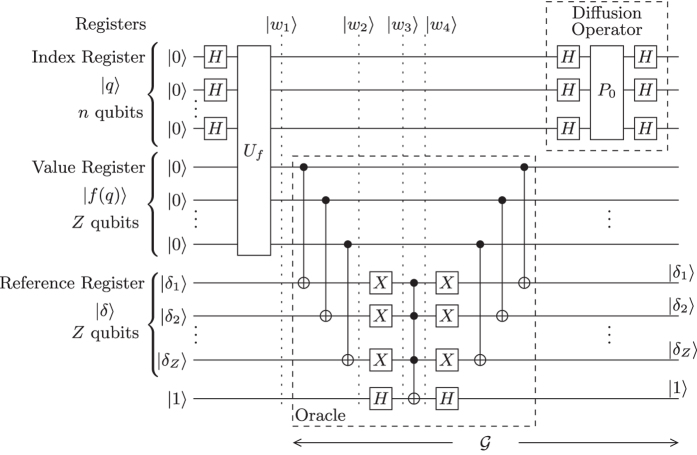
Quantum Circuit of Grover’s QSA, showing the initialization and a single Grover iteration, which consists of a sequential employment of the Oracle and the Diffusion operator. In this architecture, only the index register is required to be maintained in a superposition of states outside the Oracle operator.

The architecture of Grover’s operator 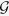 is depicted in Fig. 2. Only the index register is visible in Fig. 1. The proposed noise model of Fig. 1, in conjunction with the architecture of Fig. 2 considers all possible noise locations of the index register in the Grover circuit, as it will be further explained in the next Section.

## Imperfect Grover Quantum Search

If perturbations are imposed by the depolarizing channels on the quantum circuit of Fig. 1, each qubit may be affected by a bit flip termed as an *X*-error, a phase flip termed as a *Z*-error, or both a phase and a bit flip termed as a *Y* = *XZ*-error^4^. The depolarizing channel that models the depolarizing effects inflicts one of the three aforementioned qubit errors independently upon each qubit with a probability of *p*/3, where *p* ∈ [0, 1] is the depolarizing probability of the channel. If a qubit remains unaffected by the channel with a probability of (1 − *p*), it may be described as if the identity operator *I* was applied to it.

**Figure 3:**
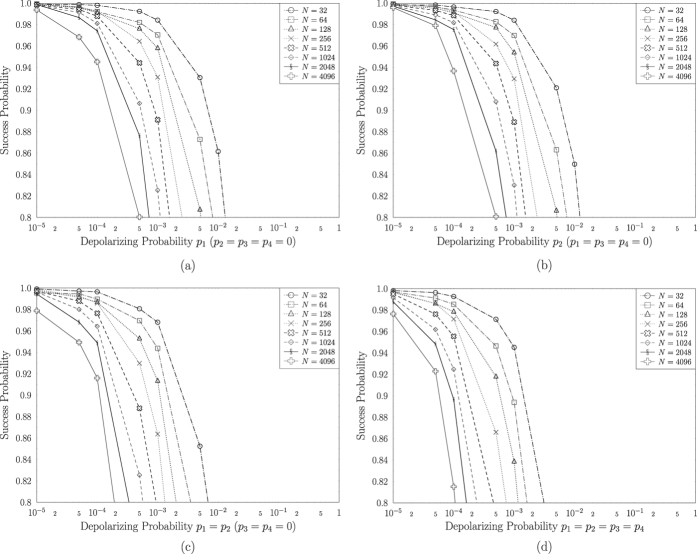
Success probability of Grover’s QSA, when depolarizing channels occur (**a**) only before the Oracle, (**b**) only right after the Oracle, (**c**) only before and right after the Oracle, (**d**) in all locations on Grover’s operator’s circuit, shown in Fig. 1, with respect to the depolarizing probability of the channels, when the architecture of Fig. 2 was used. Randomly generated databases with different sizes *N* were used, while a single solution *S* = 1 was present in a random position in the database in each search problem.

The fact that the quantum perturbations may occur independently on each of the 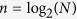 qubits is translated in two or more quantum states, if *S* > 1, that represent solutions having a different amplitude at any point. Similarly, two or more quantum states that are not solutions may have a different amplitude and therefore a different probability to be observed. Figure 3 depicts the success probability of Grover’s QSA, when a realistic practical circuit is subjected to a depolarizing channel, where [*p*_1_, *p*_2_, *p*_3_, *p*_4_] corresponds to the depolarizing probabilities at four different locations in the circuit, as stated in Fig. 1, when the architecture of Fig. 2 is used. More specifically, we have introduced four different scenarios, where the depolarizing channels perturb the system only before the Oracle, associated with [*p*_1_, 0, 0, 0]; only after the Oracle and before the first Hadamard gate, associated with [0, *p*_2_, 0, 0]; both right before and right after the Oracle, associated with [*p*_1_, *p*_2_, 0, 0]; and finally, in every possible location, associated with [*p*_1_, *p*_2_, *p*_3_, *p*_4_]. Since the index register remains unaltered during a *U*_*f*_ operation, any bit flip or phase flip that may occur on the index register between the two *U*_*f*_ gates of Fig. 2, may be equivalently modelled as an error on the index register occurring after the Oracle and before the diffusion operator. The index register may be stored in a quantum memory^42^, where quantum stabilizer codes are periodically employed, until the Oracle operation has been completed. A similar procedure may be applied for the value register, when the diffusion operator is applied to the index register. It should be noted that in every scenario of this contribution, all the non-zero depolarizing probabilities are set to be equal to each other, for the simplicity of presentation. In practice, the depolarizing probabilities may differ. A step-by-step example is provided in Supplementary Section 5. In Fig. 3, we have employed Grover’s QSA to various randomly generated databases having different sizes *N*, for a sufficiently high number of times. All databases have a single solution *S* = 1, represented by a randomly selected quantum state. In each of the scenarios, Grover’s QSA was stopped after *L*_*opt*_ number of iterations, as if it was based on an ideal, perturbation-free circuit, where *L*_*opt*_ was calculated according to (2) for a database size *N* and for *S* = 1 solution.

We may observe in Fig. 3 that the success probability is degraded when the size of the database *N* is increased, regardless of the specific locations of the depolarizing perturbations. This is expected, since when the search is performed in a larger database, more qubits are involved, therefore more errors will occur and due to the error propagation in the circuit, the success probability will be reduced. At the same time, the optimal number *L*_*opt*_ of iterations is higher in databases having higher size *N*, as encapsulated in (2), while keeping the number of solutions *S* fixed, allowing more depolarizing errors to affect the qubits and subsequently to reduce Grover’s QSA’s success probability. By comparing Fig. 3a to Fig. 3b, the success probability of Grover’s QSA is seen to be similar for the same database size, indicating that the presence of depolarizing perturbations right before or right after the Oracle imposes a similar performance degradation on Grover’s QSA. However, if depolarizing is inflicted both right before and right after the Oracle, the success probability of Grover’s QSA erodes, as illustrated in Fig. 3c, due to the error propagation within the quantum circuit. This is also verified by Fig. 3d, where a depolarizing perturbations is imposed at every possible location of Fig. 1, resulting in a degraded performance, when compared to the other three scenarios.

**Figure 4:**
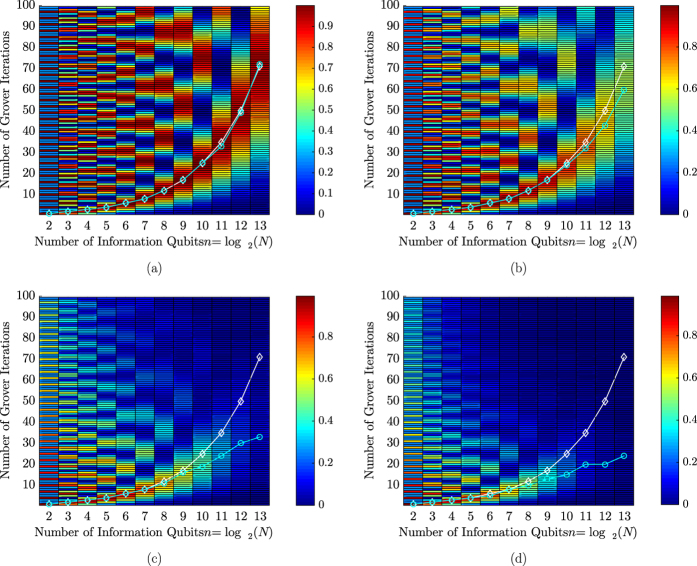
Success probability of Grover’s QSA, when a depolarizing channel appears only right before the Oracle in Grover’s operator’s circuit, shown in Fig. 1, with respect to the depolarizing probability of the channel (**a**) p_1_ = 10^−4^, (**b**) p_1_ = 10^−3^, (**c**) p_1_ = 5 · 10^−3^, (**d**) p_1_ = 10^−2^, the size of the database *N* and the number of Grover iterations *L*. The circuit architecture of Fig. 2 was employed. Randomly generated databases with different sizes *N* were used, while a single solution *S* = 1 was present in a random position in the database in each search problem. The white curve marked by the diamonds indicates the optimal number of Grover iterations *L*_*opt*_ that would be required in the ideal Grover’s QSA, associated with *p*_1_ = 0, while the cyan curve marked by the circles represents the actual optimal number of Grover iterations for the specific value of depolarizing probability *p*_1_.

Figure 4 presents the success probability of Grover’s QSA for various database sizes and for diverse values of the depolarizing probability *p*_1_, when quantum perturbations are only imposed right before the Oracle operator during each Grover iteration, in the architecture of Fig. 2. Each search problem includes a single solution associated with *S* = 1, randomly placed in the database. It may be observed that the inherent periodicity of Grover’s QSA with respect to the number *L* of applying Grover’s operator 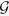 is lost, while increasing the depolarizing probability, due to the associated error propagation, which becomes more and more dominant as more depolarizing perturbations are imposed on the search process. Once again, the effect that quantum perturbations have on larger databases is more catastrophic, than that inflicted upon smaller databases, since more error-prone qubits are employed. It is worth mentioning that according to Fig. 4a,b,c and d, when a depolarizing channel is introduced, the optimal number *L*_*opt*_ of Grover iterations, required for maximizing the success probability becomes lower upon increasing the depolarizing probability *p*_1_. At the same time though, the maximum success probability that corresponds to *L*_*opt*_ is reduced as *p*_1_ is increased.

## Quantum Error Correction in Grover’s Quantum Search Algorithm

In order to improve the performance of Grover’s QSA, when quantum perturbations are present in the quantum circuit, quantum error correction codes may be employed. The quantum error correction codes may impose redundancy on the information, or *logical* qubits, at the positions in the circuit of Fig. 1, where quantum noise may appear and then flawlessly decode by correcting the quantum flips before the application of the subsequent unitary operator of the quantum circuit. For example, in the quantum circuit of Grover’s operator in Fig. 1, quantum error correction codes may be used for encoding and decoding the information qubits, which will eventually be observed between each unitary operator. Naturally, since encoding and decoding quantum information itself needs unitary operators, the exact locations of where it is beneficial to include quantum error correction codes for stabilizing the quantum information states will be decided based on the technology selected for implementing quantum computers. In our proposed model, we have opted for introducing depolarizing perturbations between the main unitary operators of Grover’s QSA for clarity and for ease of demonstrating its benefits, while stating that in practice quantum noise may also be present within the quantum operators, such as the Oracle operator for instance.

In general, quantum turbo codes^23^,^24^,^25^, quantum low density parity check codes^26^,^27^,^28^ and quantum polar codes^29^,^30^, require a long stream of information qubits for achieving their full potential. Since the 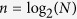 information qubits of Grover’s QSA, presented in Fig. 1, are used simultaneously in parallel by each quantum gate, quantum codes that operate well with a short information qubit stream, such as Steane code^31^ and QBCH codes^32^,^33^,^34^, may be better suited for combating quantum perturbations in quantum search algorithms. Both the Steane code as well as the QBCH codes belong to the family of stabilizer codes^44^,^45^, which is a generalized formalism for designing quantum codes from the known family of classical codes. The encoding circuit of the QBCH code is designed using the methodology of ref. 46, which is detailed in the Methods Section. Usually, the encoding and decoding circuits are assumed to be fault-tolerant^35^,^42^,^47^. Assuming erroneous encoders or decoders, the noise imposed may be represented by appropriately adjusting the depolarizing probabilities of the noise model.

**Figure 5:**
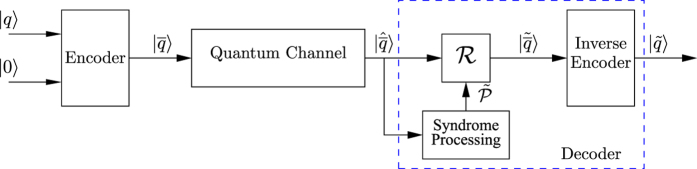
General schematic of a quantum system employing a stabilizer code.

Figure 5 shows the general schematic of a quantum system relying on a stabilizer code. An [n,k] stabilizer code maps a k-qubit information word, or k *logical* qubits, |*q*〉 onto an n-qubit codeword, or n *physical* qubits, 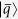 with the aid of (n − k) ancillary qubits initialized to the state |0〉. Furthermore, if 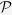 is the channel error inflicted on the codewords, then 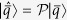 is the noisy codeword received at the decoder. A 3-step decoding process is then invoked for recovering the intended transmitted information 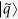, as described in the Methods Section and exemplified in Supplementary Section 6 with the aid of a step-by-step example.

## Employment of Stabilizer Codes in Grover’s Algorithm

**Figure 6:**
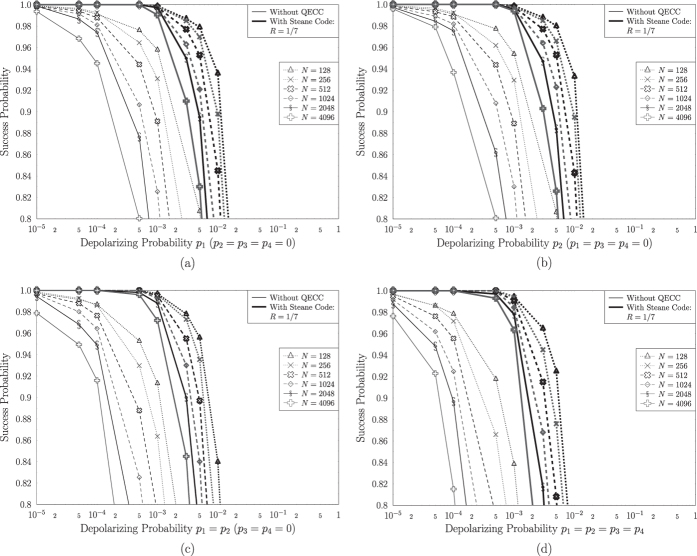
Success probability of Grover’s QSA, when quantum noise occurs (**a**) only before the Oracle, (**b**) only right after the Oracle, (**c**) only before and right after the Oracle, (**d**) in all locations on Grover’s operator’s circuit, shown in Fig. 1, with respect to the depolarizing probability of the channels, when the architecture of Fig. 2 was used. Steane code with rate *R* = 1/7 has been employed for improving the performance of Grover’s QSA. Randomly generated databases with different sizes *N* were used, while a single solution *S* = 1 was present in a random position in the database in each search problem.

In Fig. 6 we characterize the performance of Grover’s QSA, in terms of the probability of successfully finding the single solution, when the Steane code having a coding rate of *R* = 1/7 is employed in the specific locations, where depolarizing perturbations appear in the architecture of Fig. 2. Four different scenarios have been investigated for various database sizes *N*, while including only a single solution associated with *S* = 1 in each search. We may observe that regardless of the locations of the depolarizing perturbations, the Steane code improves the performance of Grover’s QSA. Quantitatively, Steane code is capable of correcting quantum errors, when the depolarizing probability is equal to or lower than approximately 5 ⋅ 10^−2^. The Quantum Bit Error Ratio (QBER) improvement offered by the Steane code is increased, when the depolarizing probability is reduced, since the probability of overwhelming this single-error correcting code is reduced, which would result in correcting the wrong qubits, thereby inflicting extra errors. This is the reason why in Fig. 6 the improvement is more significant, when larger databases, associated with higher *N* values, are used. For example, based on Fig. 6d, when searching in a database of *N* = 4096, the system employing the Steane code may achieve a success probability of 0.98 for depolarizing probabilities 80 times higher than those required by the uncoded quantum search for achieving the same probability of success.

Similarly to their uncoded counterparts, the quantum systems that employ quantum error correction during the quantum search are affected by the quantum perturbations within the quantum circuit. More precisely, as we may observe in Fig. 6a and b, which correspond to the scenarios, where depolarizing may occur only right before or only right after the Oracle operator, respectively, they exhibit an equivalent performance. However, when quantum noise is present in both the aforementioned locations, according to Fig. 6c the success probability is reduced, even when quantum error correction is used. Finally, based on Fig. 6d, as expected due to error propagation, a degraded performance is achieved, when depolarizing perturbations occur everywhere in Grover’s QSA circuit of Fig. 1.

**Figure 7:**
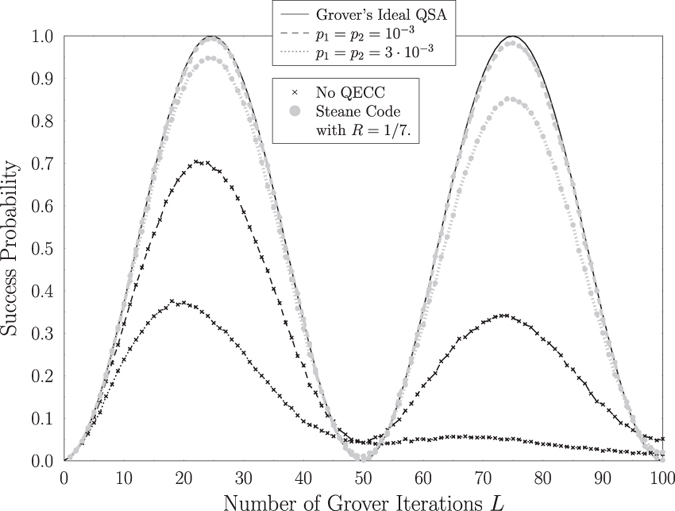
Success probability of Grover’s QSA in a database with *N* = 1024 entries and *S* = 1 solution, with respect to the number of applications *L* of Grover’s operator 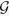, when depolarizing channels appear before and after the Oracle operator of Fig. 1, associated with *p*_3_ = *p*_4_ = 0. The architecture of Fig. 2 is used. Two different depolarizing probabilities are assumed, namely *p*_1_ = *p*_2_ = 0.001 and *p*_1_ = *p*_2_ = 0.003, while the performance when Steane code is employed for cancelling the channels’ effects is compared to that of an uncoded system. The performance of the ideal Grover’s QSA is also attached as a reference.

In order to delve deeper into the intuition of depolarizing in Grover’s QSA’s circuit, let us investigate a scenario, where we have a database of size *N* = 1024 and *S* = 1 solution, which may be considered as the “worst-case” scenario, since according to (2) the maximum possible number of Grover iterations will be required for a fixed database size, when *S* = 1. Let us also assume that quantum perturbations may affect the qubits right before or right after the Oracle operation of Fig. 1, associated with *p*_3_ = *p*_4_ = 0. The curve of Grover’s ideal QSA seen in Fig. 7 represents the upper limit of the achievable performance for each application of Grover’s operator, since it corresponds to the case, where *p*_1_ = *p*_2_ = *p*_3_ = *p*_4_ = 0. It should be noted that in this scenario the optimal number of Grover iterations, given by (2), is equal to *L*_*opt*_ = 25. According to Fig. 7, when the quantum noise affects a qubit with a probability of *p*_1_ = *p*_2_ = 10^−3^, the performance of the quantum search employing Steane’s code for quantum error correction is near-optimal, reaching a success probability of 99.3% at *L* = 25 Grover iterations, indicating the ability of Steane’s code to correct both the bit and phase flips introduced by the depolarizing perturbations. On the other hand, if no quantum error correction was employed for mitigating the effects of the depolarizing perturbations, the maximum achievable probability of success is equal to 66.7% at *L* = 24 Grover iterations. The performance degradation becomes higher, when the depolarizing probability is increased. Still based on Fig. 7, if we have say *p*_1_ = *p*_2_ = 3 ⋅ 10^−3^, then by using Steane’s code we may reach a maximum success probability of 95.3% at *L* = 25 Grover iterations, instead of an inferior 33.3%, which would be the case if no quantum error correction was employed.

Since the QBCH[15, 7] is a block code, it may be used in a database associated with *n* = 7 information qubits, which results in a database with *N* = 2^*n*^ = 128 entries, so that all the qubits participate in the same encoding process. Even though the performance is expected to be worse than that of the Steane code, the coding rate of the QBCH[15, 7] code is higher than that of Steane code’s. More specifically, the QBCH[15, 7] has a coding rate of *R*_*QBCH*[15,7]_ = 7/15 = 0.47, while Steane’s code has a coding rate of *R*_*Steane*_ = 1/7 = 0.14. A QBCH[n, k] code associated with k logical qubits may also be employed in databases (*n*/k) times in parallel, where the number of information qubits *n* is a multiple of its number of logical qubits k. For instance, the QBCH[15, 7] code may be used (*n*/k) = 2 times in parallel for Grover’s QSA in databases associated with *n* = 14, which is translated into databases having a size of *N* = 2^*n*^ = 16384, encoding the first *k* = 7 qubits with each other, as well as the last *n* − *k* = 7 qubits with each other. Furthermore, the QBCH[15, 7] code may be combined with Steane’s code for providing a hybrid combination of quantum error correction. For example, if we have a database associated with *N* = 1024 entries, we require *n* = 10 qubits in the index register. The QBCH[15, 7] code may be used for encoding the first 7 qubits, while the last 3 qubits may be encoded using Steane’s code, requiring 15 + 7 ⋅ 3 = 41 physical qubits in total, instead of the 70 qubits that would be required by exclusively using Steane’s code. However, a degraded performance is expected, when compared to using only Steane’s code. Naturally, if a universal quantum computer processes multiple quantum algorithms simultaneously, information qubits of different processes may be encoded using the same quantum code for stabilizing purposes, if the timing allows it.

**Figure 8:**
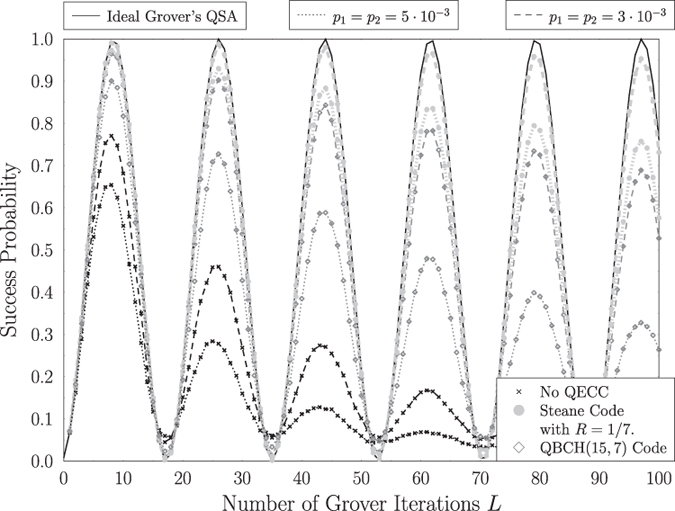
Success probability of Grover’s QSA in a database with *N* = 128 entries and *S* = 1 solution, with respect to the number of applications *L* of Grover’s operator 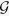, when depolarizing channels exist before and after the Oracle operator of Fig. 1, associated with *p*_3_ = *p*_4_ = 0, when the architecture of Fig. 2 is used. Two different depolarizing probabilities are assumed, namely *p*_1_ = *p*_2_ = 0.003 and *p*_1_ = *p*_2_ = 0.005, while the performance when QBCH[15, 7] code is employed for cancelling the channels’ effects is compared to that of the Steane code, as well as that of an uncoded system. The performance of the ideal Grover’s QSA is also attached as a reference.

Let us compare the influence that the QBCH[15, 7] code has on Grover’s QSA to that of Steane’s code, when a quantum search is performed in a database having *N* = 2^*n*^ = 128 entries, where a single solution *S* = 1 is present. Based on Fig. 1, we opted for a system, where quantum perturbations may only be present only before or after the Oracle operator, represented as *p*_3_ = *p*_4_ = 0. In more detail, we investigate two scenarios, where *p*_1_ = *p*_2_ = 0.003 and *p*_1_ = *p*_2_ = 0.005. The success probability of Grover’s QSA when no quantum error correction is used in both scenarios, as well as when the QBCH[15, 7] and Steane’s code are employed, may be seen in Fig. 8. As expected, Steane’s code offers an improved performance, when compared to the QBCH[15, 7] code, since it achieves a success probability of 99.1% and 97.3% for *p*_1_ = *p*_2_ = 0.003 and *p*_1_ = *p*_2_ = 0.005, respectively, after *L*_*opt*_ = 8 according to (2), while the QBCH[15, 7] code yields a success probability of 96.5% and 90.1% for *p*_1_ = *p*_2_ = 0.003 and *p*_1_ = *p*_2_ = 0.005, respectively, after the same number of Grover iterations. At the same time though, the QBCH[15, 7] code requires only 8 auxiliary qubits for encoding the 7 information qubits, resulting in a coding rate of *R* = 7/15, while again Steane’s code requires 42 auxiliary qubits, due to its low coding rate of *R* = 1/7. Therefore, a trade-off between performance and available resources is unavoidable in this case. Still based on Fig. 8, Grover’s QSA performs better when a quantum error correction code is used, and its probability of success is reduced, when we increase the number of Grover iterations, due to the error propagation in the information qubits.

In every scenario of Grover’s imperfect QSA, the error propagation eventually results in the termination of the periodicity of Grover’s QSA’s success probability with respect to the number of Grover iterations as demonstrated in Fig. 4, by initially reducing the peak of the success probability and finally making it equivalent to a random guess. The speed of this phenomenon is increased, when a higher depolarizing probability is present in the quantum system, as depicted in Fig. 7.

## Conclusions and Open Problems

In this treatise, we demonstrated that quantum error correction codes, which do not require long information qubit streams, may be employed for stabilizing the information qubits of quantum search algorithms. We presented a model of Grover’s QSA in Fig. 1, where quantum perturbations may occur in different locations of its quantum circuit, modelled in the form of depolarizing channels, and evaluated its probability of success as a function of the number of Grover iterations, as well as that of the depolarizing probabilities of the existing channels. We also analysed and investigated a circuit architecture devised for Grover’s QSA. In practice, the specific technologies used for creating a quantum computer will determine the particular locations, where it is beneficial to stabilize the qubits of a quantum algorithm, as well as the suitable circuit architectures. The methodologies we presented here are applicable to any arbitrary positions in the circuit of Grover’s QSA.

Furthermore, the effect that depolarizing has on the optimal number of Grover iterations was also investigated in Fig. 4. Moreover, we managed to achieve a depolarizing probability improvement higher than an order of magnitude that may be tolerated, when aiming for a ~100% probability of success in large databases, when using Steane’s code for correcting the quantum errors, as illustrated in Fig. 6. According to Fig. 8, the near-half-rate QBCH codes may also improve the performance of Grover’s QSA, but as expected, they offer a lower gain, than that offered by Steane’s code, again at a higher coding rate.

As future research, it may be beneficial to investigate the effect that depolarizing may have on other quantum search algorithms, variants of Grover’s QSA, such as the Boyer-Brassard-Hø yer-Tapp (BBHT) QSA^43^, or the Dürr-Hø yer algorithm^48^, which are based on Grover’s QSA, but may prove to be more resilient to bit and phase errors due to their pseudo-random methodology. In addition, an impactful research topic would be to investigate the performance of quantum search algorithms, when depolarizing perturbations occur within the quantum operators, such as the Oracle. Furthermore, more complex quantum error correction codes, such as the Quantum Low-Density Parity-Check (QLDPC) codes^28^,^49^, or the Quantum Turbo Code (QTC)^23^,^25^,^50^, may be employed for operating close to the Hashing bound in large-scale quantum systems, where multiple quantum computing algorithms will be running in parallel, therefore a plethora of qubits exploited by different algorithms will have to be stabilized simultaneously, assuming a central controller will perform the necessary synchronization.

## Methods

### Initialization and Oracle Circuit in Grover’s QSA

The operation of both Grover’s operator and of the Oracle^51^ is illustrated in Fig. 2. The *index register* |*q*〉 consists of 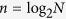 qubits and it is initialised to an equiprobable superposition of all legitimate states, as described in (1). When the quantum search algorithm is employed for searching through the values of a function *f(q*), then the unitary quantum gate *U*_*f*_ that computes this function is applied both to the n-qubit index register |*q*〉, as well as to the *Z*-qubit *value register* |*f(q*)〉, which is initialized to the all-zero state. The operation of *U*_*f*_ leaves the index register |*q*〉 unaltered, while entangling the qubits of the index register and of the value register with each other, hence resulting in the state


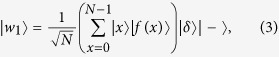


where |*δ*〉 is the *reference register* and 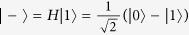 is an auxiliary qubit. In other words, each legitimate input *x* of the function *f*(⋅) is entangled with its respective value *f(x*). The auxiliary reference register |*δ*〉 also consists of *Z* number of qubits, since it will be compared to the value register |*f(q*)〉. Please note that as mentioned in the context of (3), the reference register is not found in a superposition of states, but instead is set to the reference value *δ* that we are looking for. Since we employ Grover’s QSA in our scenario, Fig. 2 depicts the Oracle that searches for the specific value |*f(x*)〉 that is equal to |*δ*〉. In order to achieve this, *Z* number of Controlled-NOT (CNOT) gates^4^ are used in conjunction with the qubits of the value register acting as the control qubits, while those of the reference register represent the target qubits. During the operation of a CNOT gate, the state of the target qubit is flipped, when the control qubit is equal to |1〉.

Following the operation of the CNOT gates, the reference register will be entangled to the value register. Additionally, the value of the reference register’s qubits returned for the specific solution state |*x*_*s*_〉, for which we have *f(x*_*s*_) = *δ*, will always be |0〉^⊗*Z*^. In order to further clarify this, we have considered a brief example (see Supplementary Section 7). In other words, the Oracle marks the particular solution in the index register by flipping its sign with the aid of a concerted action by a value register, a reference register and an additional auxiliary qubit. Then, the inverse operation is performed for the sake of removing the entanglement between the value register and the reference register.

In this treatise, we assume that the value register is always error-free and any perturbations occur at the index register. This is the reason why only the index register is illustrated in Fig. 1. The diffusion operator is applied only to the index register, as depicted in Fig. 2. The depolarizing probabilities *p*_1_ and *p*_2_ of Fig. 1 correspond to the quantum circuit of Fig. 2. An example encapsulating the process of the diffusion operator is provided in Supplementary Section 5.

If other quantum search algorithms were employed, the Oracle’s circuit would be different. For example, if we employed a variant of Grover’s QSA, as proposed by Dürr and Høyer in ref. 48, which aims for finding the minimum of a database, then both the initialization stage of Fig. 2, as well as the diffusion operator would remain the same, but the Oracle’s circuit of Fig. 2 would be replaced by a specific bit string comparator circuit, which would mark all the states that have a lower value than the reference value *δ*.

### Encoder of Quantum BCH Code

The Steane code is equivalent to the QBCH[7, 1] code. The PCM of the single error-correcting QBCH[15, 7], which is constructed from the dual-containing classical BCH(15, 11) code, is


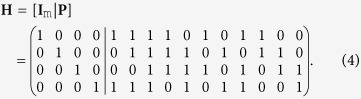


The encoding circuit of QBCH[15, 7] is derived as follows (see Supplementary Fig. 5):

- Let **H** be an m × n = 4 × 15 classical dual-containing PCM of (4). We transform **H** into the matrix 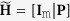 using row operations as well as column permutations. The resultant matrix **I**_m_ is an m × m identity matrix, while **P** is an m × (n − m) binary matrix. For the **H** of (4), we have 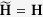.
- As a next step, we apply row operations to **P**, reducing it to 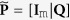, where **Q** is an m × (n − 2m) binary matrix. Therefore, we get
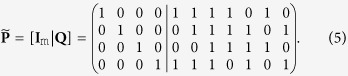
- Let 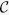 be the classical code having the PCM 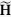 and the dual code 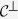, such that 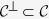. An [n, k] dual-containing CSS code maps each of the 2^k^ superimposed states of a k-qubit information word onto a unique coset of the dual code 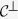 in the code space of 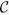. The cosets of 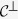 in 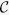 may be obtained by adding a codeword of 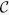 to all the codewords of 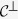. However, only those codewords of 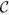 generate a unique coset of 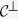, which do not differ by an element of the 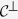. In this context, the encoder may be implemented in two steps (see Supplementary Fig. 5).
- In the first step, the matrix **Q** of (5) acts on the second block of m = 4 qubits controlled by the last k = (n − 2m) = 7 qubits, which constitute the information word. More explicitly, a CNOT gate acts on the *i*th qubit of the second block of m qubits, which is controlled by the *j*th information qubit, if *Q*_*ij*_ = 1. This may be encapsulated as follows
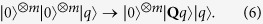
The resultant states constitute the set of codewords of 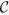, which do not differ by any element of 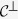 and there ore are capable of generating unique cosets of 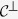.
- The second stage adds the codewords of 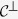 to the codewords of 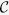 generated in the previous step. More specifically, the second stage on its own generates the codespace of 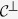 according to the PCM 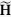. For a classical code 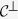, the first m bits are the systematic information bits, which can have either the value of 0 or 1. Consequently, the first m = 4 auxiliary qubits undergo a Hadamard transformation. Finally, the matrix **P** of (4) acts on the last (n − m) qubits controlled by the first m qubits for the sake of generating the codespace of 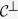. More explicitly, a CNOT gate acts on the *j*th qubit of the last (n − m) qubit, which is controlled by the *i*th qubit, if *P*_*ij*_ = 1.

### Decoding Process of Stabilizer Codes

Both the Steane code and the QBCH code, which are invoked in this treatise for improving the performance of Grover’s QSA in the presence of perturbations, are dual-containing Calderbank-Shor-Steane (CSS) type^25^ stabilizer codes. More specifically, these codes were designed from dual-containing classical codes. We derived the encoding circuit of QBCH using the method conceived by Mackay *et al*. in ref. 46. The decoding process of Fig. 5 proceeds as follows:

1. Syndrome Processing: Since any observation of a qubit perturbs its superimposed quantum state, a quantum decoder should not measure the received qubits. Therefore, inspired by the Parity Check Matrix (PCM)-based syndrome decoding of classical codes^52^, a quantum decoder circumvents the associated measurement operation by observing the error syndromes, rather than the actual quantum information. For an [n, k] stabilizer code, this is achieved by applying (n − k) commuting n-qubit Pauli operators^4^, called stabilizers, to the received codewords 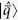. The stabilizers yield an eigenvalue of +1 for valid codewords and −1 for the corrupted ones, which corresponds to a syndrome value of 0 and 1, respectively. Analogous to the syndrome decoding of classical codes, a syndrome value of 0 marks the absence of quantum flips in a codeword, while a value of 1 denotes the presence of quantum flips. More specifically, the eigenvalue is +1 if 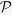 commutes with the *i*th stabilizer *g*_*i*_, while it is −1 if 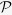 anti-commutes with the stabilizer *g*_*i*_, which may be formulated as:
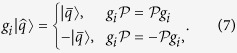
Hence, within the ‘syndrome processing’ block of Fig. 5, the decoder computes the syndrome of the received sequence 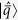 and uses the resultant syndrome sequence to estimate the perturbation-induced error pattern 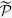 with the aid of a classical syndrome decoding process.
2. Error Recovery (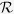): The error recovery block ‘
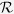
’ of Fig. 5 restores the potentially error-free coded stream using the estimated error pattern 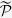.
3. Inverse Encoder: Finally, the ‘inverse encoder’ of Fig. 5 processes the recovered coded sequence 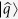, yielding the estimated original information qubits 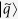. Here the terminology of “inverse encoder” is used because, in contrast to an encoder, which maps logical qubits onto the physical qubits, an inverse encoder carries out the inverse operation by mapping physical qubits onto the logical qubits. More explicitly, an inverse encoder may have the same circuit as an encoder, but operates from right (physical qubits) to left (logical qubits).

### Implementation

The systems investigated in this contribution were implemented using object oriented programming in C++, with the aid of the IT++ library, and simulated on the University’s supercomputer. Since a universal quantum computer is not available at the time of writing, Grover’s QSA was designed in the classical domain. Let us describe the steps of simulating Grover’s QSA with the aid of a classical computer. Initially, a random database of size *N* is generated. Since the number of solutions *S* in the database is expected to be known prior to the search, *S* random entries are picked in the database, and the last *S* − 1 entries change their values to that of the first randomly picked solution. That value is equal to *δ*. Therefore, an *N*-element database with *S* entries equal to *δ* has been generated. Without loss of generality, the entries in the database assume values in the range of [0, 1], while making sure that no more than *S* entries have values equal to *δ*.

The unitary operators were implemented by using their matrix representation. The quantum states were described by their vector representation. The Oracle in Grover’s QSA is also employed by using its matrix representation. Since the action of the Oracle effectively flips the phase of the specific state that is a solution, and since the simulations were performed in a classical computer, we are capable of finding the specific entries that represent solutions by performing a full search. The optimal number of Grover iterations *L*_*opt*_ is calculated before the initiation of the search according to (2).

The measurement of a quantum state was programmed by generating a uniformly-distributed random number in the [0, 1] range and searching which specific part of the cumulative sum of the measured state’s probabilities it is found in. The particular quantum state that corresponds to the probability range that the randomly generated number belongs to is assumed to be the observed state.

Quantum error correction codes were emulated using the Heisenberg representation of the Gottesman-Knill theorem^53^. Explicitly, an n-qubit Clifford encoder, acting on a 2^n^-dimensional Hilbert space, has a 2^n^ × 2^n^ unitary matrix, which defines the evolution of the associated n-qubit system By contrast, the Gottesman-Knill theorem^53^ facilitates efficient classical simulation of the 2^n^ × 2^n^ matrix by specifying the action of the encoder under conjugation on the Pauli *X* and *Z* operators acting on each of the n qubits. Consequently, the operation of a Clifford encoder may be completely described by only tracking the evolution of the 2n operators {*Z*_1_, *Z*_2_, …, *Z*_*n*_, *X*_1_, *X*_2_, …, *X*_n_}, where *Z*_*j*_ and *X*_*j*_ represents the Pauli *Z* and *X* operator, respectively, acting on the *j*th qubit and the identity *I* on all other qubits. Furthermore, each of the 2n operators may be represented by (2n + 1) classical bits, so that two classical bits are used for mapping each Pauli operator as follows


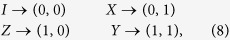


while one bit is used for the phase. However, the encoders, which differ only through a global phase, have the same impact under conjugation. Therefore, the bit used for denoting phase can be ignored and the n-qubit encoder may be characterized by a (2n × 2n) binary matrix. Based on the resultant classical representation of the quantum codes, we classically simulated the performance of the system of Fig. 5 by following the evolution of the channel error induced as follows:

1. Quantum Channel: Recall that a quantum depolarizing channel, characterized by the probability *p*, inflicts either a bit-flip or a phase-flip or in fact both with a probability of *p*/3. Therefore, according to the Pauli-to-binary mapping of (8), the quantum depolarizing channel reduces to two Binary Symmetric Channels (BSCs), one channel for the phase errors and the other for the bit errors, each having a crossover probability of 2*p*/3. Consequently, we simulated the quantum channel by generating two independent BSCs, yielding the classical error patterns *P*_*z*_ and *P*_*x*_ for phase and bit errors, respectively.
2. Syndrome Processing: The resultant error patterns *P*_*z*_ and *P*_*x*_ of the two BSCs were fed independently to a classical syndrome decoder for estimating the channel-induced classical errors 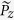 and 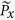.
3. Error Recovery: Then the error recovery operation of Fig. 5 was emulated by the modulo 2 addition of *P*_*z*_ and 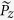, and similarly the modulo 2 addition of *P*_*x*_ and 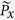, which yielded the residual phase and bit error, respectively, on the recovered output 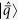 of Fig. 5. This error recovery process may be encapsulated as
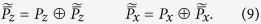
4. Inverse Encoding: Let 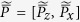 be the 2n-bit residual error imposed on the recovered physical qubits 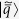. Let us assume furthermore that *V* is the equivalent (2n × 2n) binary matrix of the n-qubit quantum code 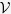. Based on this notation, passing the residual error through the inverse encoder *V*^−1^ yields

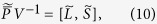


where 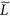 represents the residual error imposed on the logical qubits 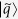 of Fig. 5, while 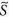 denotes the residual error on the auxiliary qubits, which were initialized to |0〉 at the input of the encoder of Fig. 5. Hence, we applied the error 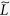 to our intended logical qubits |*q*〉 of Fig. 5 to get the estimated logical qubits 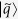 of Fig. 5.

## Additional Information

**How to cite this article**: Botsinis, P. *et al*. Quantum Error Correction Protects Quantum Search Algorithms Against Decoherence. *Sci. Rep.* **6**, 38095; doi: 10.1038/srep38095 (2016).

**Publisher's note:** Springer Nature remains neutral with regard to jurisdictional claims in published maps and institutional affiliations.

## Supplementary Material

Supplementary Information
