# Supplementary material for: Quantum Error Correction Protects Quantum Search Algorithms Against Decoherence
Source: Sci Rep. 2016 Dec 7;6:38095. doi: 10.1038/srep38095 (PMC5141447; doi:10.1038/srep38095)
Supplement: Supplementary Information [file srep38095-s1.pdf]

Quantum Error Correction Protects Quantum Search Algorithms Against Decoherence

Panagiotis Botsinis[1], Zunaira Babar[1], Dimitrios Alanis[1], Daryus Chandra[1], Hung Nguyen[1], Soon Xin Ng[1], and Lajos Hanzo[1,*]

[1]Southampton Wireless, School of Electronics and Computer Science, University of Southampton, United Kingdom
[*]Corresponding Author: lh@ecs.soton.ac.uk

1 Supplementary Figures

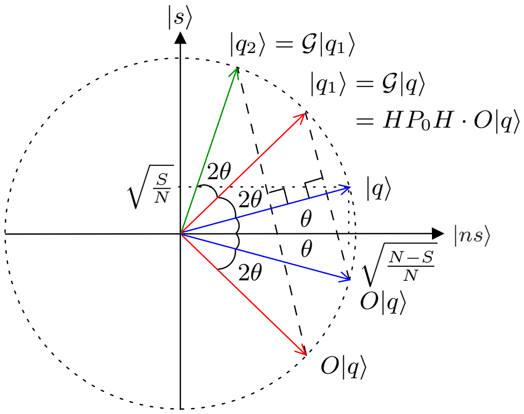

Figure 1. Two applications of Grover's operator $\mathscr{G}$ to an equiprobable superposition of all legitimate states $|q\rangle$, when no depolarizing errors are present.

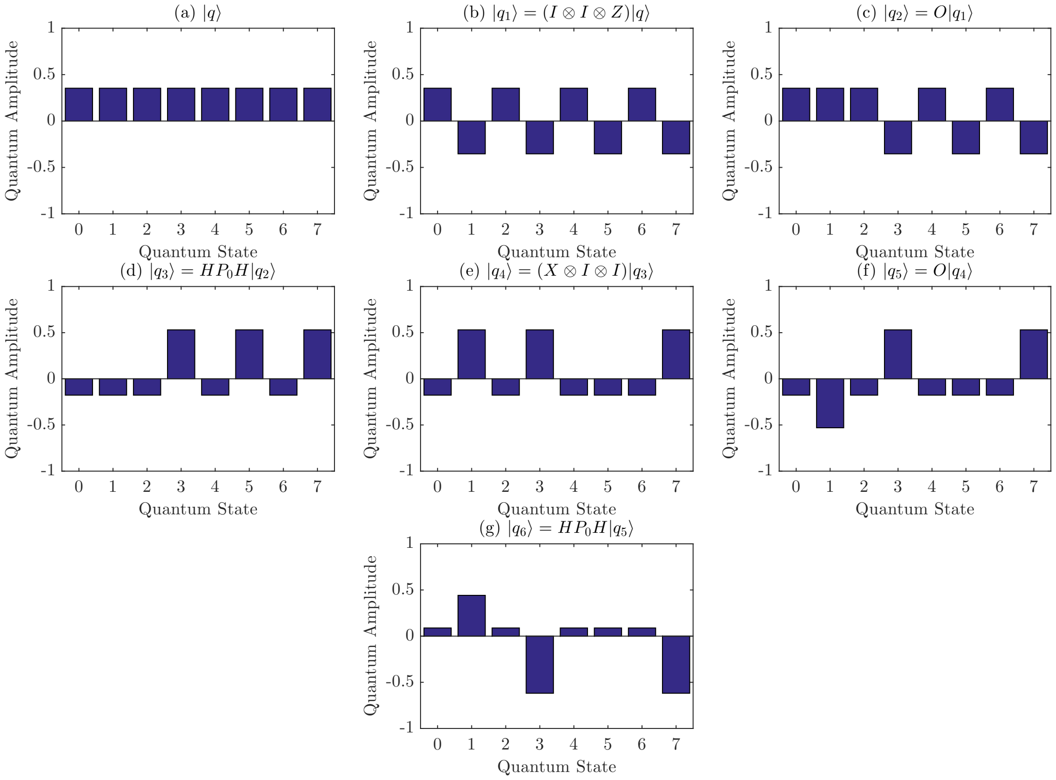

Figure 2. Example of the evolution of quantum states, when quantum perturbation occurs before the Oracle, for a database of size $N = 8$, with $S = 1$ solution present at the quantum state $|1\rangle = |001\rangle$. The optimal number of Grover iterations is $L_{opt} = 2$. A phase flip (Z error) occurs at the third qubit during the first application of Grover's operator, while a bit flip (X error) takes place for the first qubit during the second application of Grover's operator.

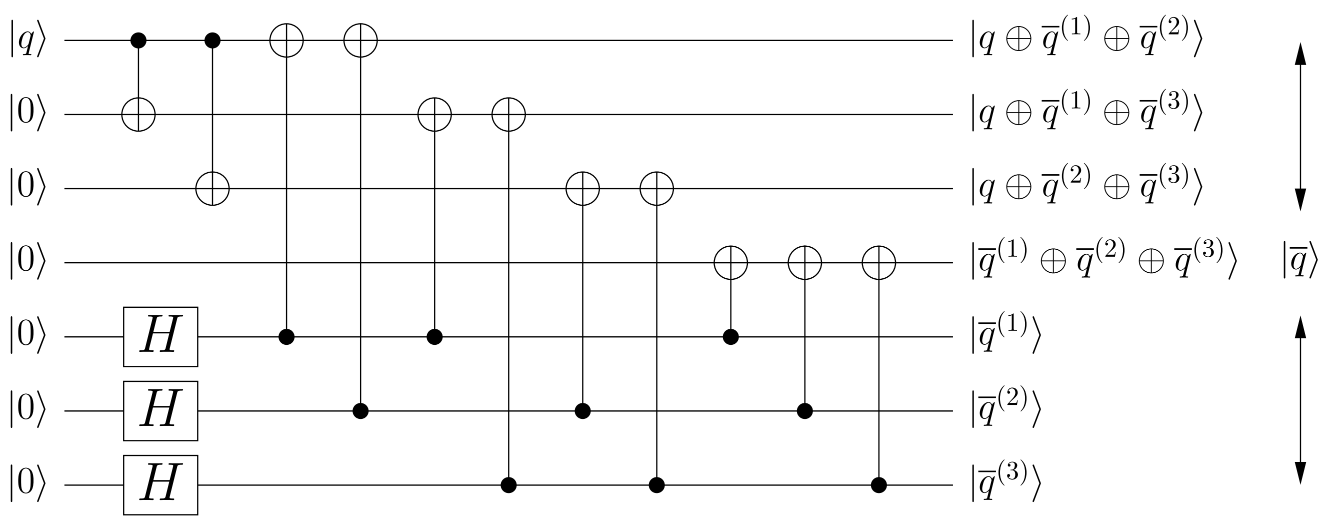

Figure 3. Encoder of Steane code.

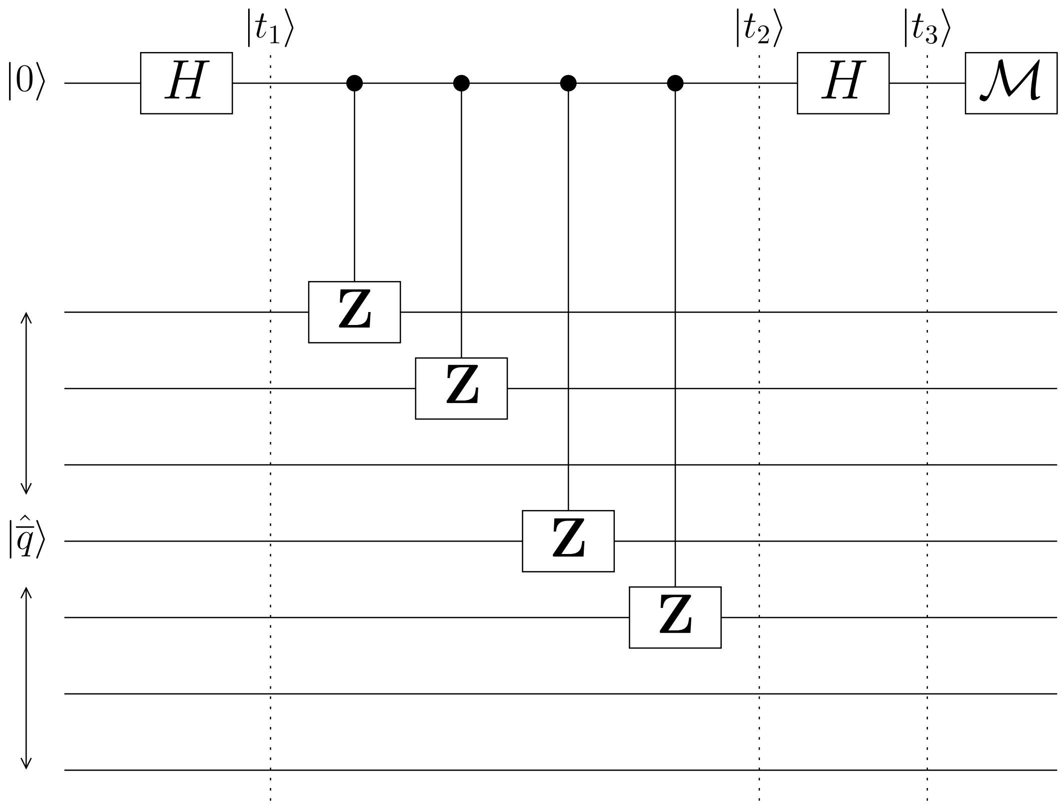

Figure 4. Implementation of the stabilizer generator $g_1 = ZZIZZII$, where $|\hat{\bar{q}}\rangle$ is the received signal from the quantum channel as seen in Fig. 6. $\mathscr{M}$ represents the measurement operation. The controlled-Z gates are replaced by the controlled-X gates for X stabilizers.

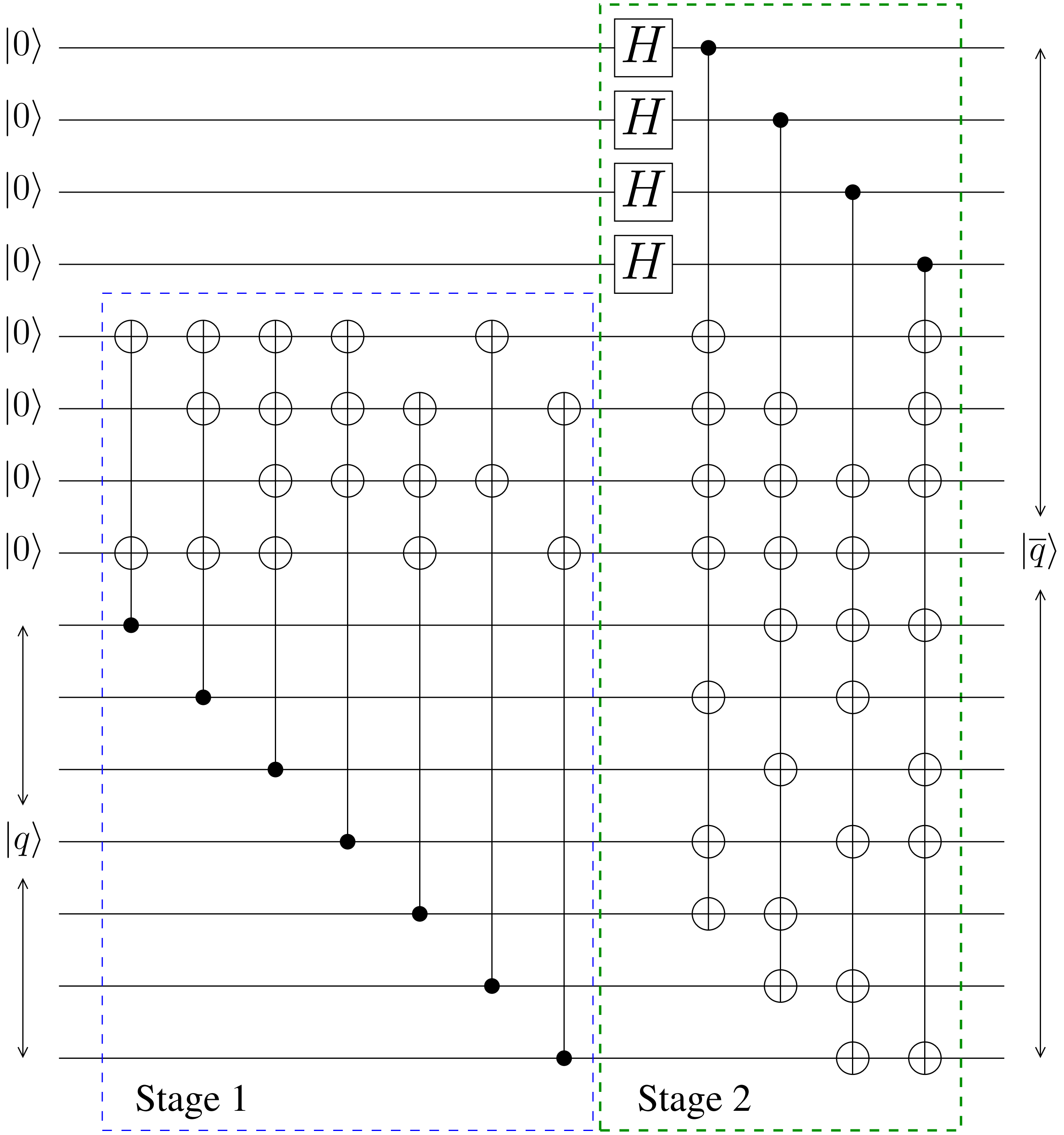

Figure 5. Encoder of QBCH$[15,7]$.

2 Supplementary Tables

Table 1. Example Function Values

Index x	0	1	2	3	4	5	6	7
Index x (bin)	000	001	010	011	100	101	110	111
Value $f(x)$	011	010	111	000	111	110	011	001
δ	010	010	010	010	010	010	010	010

Table 2. Stabilizers of the Steane code.

	**Stabilizer**
g_1	ZZIZZII
g_2	ZIZZIZI
g_3	IZZZIIZ
g_4	XXIXXII
g_5	XIXXIXI
g_6	IXXXIIX

Table 3. Look-up table of the Steane code. For bit flips, the syndrome corresponds to the generators $[g_1\ g_2\ g_3]$, while for phase errors, the syndrome is given by $[g_4\ g_5\ g_6]$.

Syndrome	Index of error
[001]	7
[010]	6
[011]	3
[100]	5
[101]	2
[110]	1
[111]	4

Table 4. Stabilizers of QBCH$[15,7]$.

	Stabilizer
g_1	ZIIIZZZZIZIZZII
g_2	IZIIIZZZZIZIZZI
g_3	IIZIIIZZZZIZIZZ
g_4	IIIZZZZIZIZZIIZ
g_5	XIIIXXXXIXIXXII
g_6	IXIIIXXXXIXIXXI
g_7	IIXIIIXXXXIXIXX
g_8	IIIXXXXIXIXXIIX

3 Applications of Quantum Search Algorithms

In the uplink of wireless communications systems, the base station employs the classic Multi-User Detection (MUD) process, which determines the specific legitimate symbols transmitted by each of the supported users. In lightly loaded, or full-rank systems, where the sum of transmit antennas of all users is lower than or equal to the number of receive antennas at the base station, low-complexity linear MUDs exhibit an adequate performance. However, in rank-deficient systems, where the number of transmit antennas is higher than the number of receive antennas, sophisticated non-linear search techniques have to be employed. The optimal classic MUD is the maximum likelihood MUD, which performs a full search for finding the specific combination of legitimate symbols that minimizes the mean squared error criterion. As demonstrated in[1–5] the specific variant of Grover's QSA proposed by Dürr and Høyer in[6] for finding the minimum in a database may be exploited in the MUD application considered.

The cluster analysis technique[7] may also be improved by Grover's QSA, which may be used for the vector quantization of the channels in wireless communications[8]. Clustering is also used in unsupervised machine learning used for data mining[9], as well as for diverse other applications, such as Gait Recognition[10,11]. With the aid of Grover's QSA, a quantum-assisted k-means or k-median algorithm has been developed[12,13] for performing low-complexity clustering. Furthermore, Grover's QSA may also be used in the field of image compression, as proposed in[14] for achieving low-complexity fractal image compression, while removing the requirement of pre-processing tools, as well as for feature extraction from medical images[15]. Quantum search may also aid in computational topology and geometrical analysis of data, such as the calculation of the Betti numbers of a data generating pattern[16].

Grover's QSA as well as its variants may also be combined with classical heuristic evolutionary algorithms, such as Genetic Algorithm[17] or Particle Swarm Optimization[18] for yielding a more potent quantum-assisted evolutionary algorithm, while imposing lower complexity than their classical counterparts. Malossini *et al.* proposed a quantum-assisted genetic algorithm[19], employing a variant of Grover's QSA in each generation of a Genetic Algorithm. The quantum-assisted evolutionary algorithms may be employed in the field of wireless communications for improving channel estimation[20,21]. To elaborate a little further, in channel estimation, a continuous-valued search algorithm may be employed for estimating both the amplitude and phase of a multipath fading channel. A carefully designed quantum-assisted evolutionary algorithm may replace the state-of-the-art classical solutions. The multi-user transmission process[22,23] may also benefit from quantum-assisted evolutionary algorithms, relying on Grover's QSA, which may be employed in the downlink of a communications system, for preprocessing the transmitted signal for eliminating the multi-user interference at the transmitter. Quantum-assisted evolutionary algorithms may outperform the classical solutions in this application.

Grover's QSA and its proposed variants may also be employed in the field of multi-objective routing, where Pareto Optimality problems appear[24]. Explicitly, when multiple contradicting objectives such as the minimum transmit power, bit error ratio and delay have to be met in routing, the specific routes, in which none of the these three parameters can be improved without degrading some of the others, belong to the optimal Pareto front. Grover's QSA may be employed for performing multiple objective based routing[25,26] at the cost of a several orders of magnitude lower complexity, when compared to the fastest classical algorithms.

4 Geometrical Representation of Grover's QSA

Supplementary Fig. 1 describes the effect that Grover's operator has on the quantum system. The x-axis represents the equiprobable superposition of the $(N-S)$ number of quantum states that are not solutions as in

$$|ns\rangle = \frac{1}{\sqrt{N-S}} \sum_{\forall i \ s.t. \ f(x_i)\neq\delta} |x_i\rangle, \tag{1}$$

while the y-axis represents the equiprobable superposition of the S states that are solutions, as encapsulated in

$$|s\rangle = \frac{1}{\sqrt{S}} \sum_{\forall j \ s.t. \ f(x_j)=\delta} |x_j\rangle. \tag{2}$$

Since the initial quantum state is constituted by an equiprobable superposition of all legitimate states, it may also be described as

$$|q\rangle = \frac{1}{\sqrt{N}} \sum_{x=0}^{N-1} |x\rangle = \sqrt{\frac{S}{N}}|s\rangle + \sqrt{\frac{N-S}{N}}|ns\rangle. \tag{3}$$

Therefore, the vector representing this initial quantum state $|q\rangle$ will have an angle of

$$\theta = \arcsin\left(\sqrt{\frac{S}{N}}\right) \tag{4}$$

with respect to the x-axis in Supplementary Fig. 1. When the Oracle is applied to the quantum system, the signs of all S quantum states that represent solutions will be flipped. This may be considered as a reflection with respect to $|ns\rangle$ as it may be verified in Supplementary Fig. 1, since all the quantum states that are not solutions remain unaltered. The diffusion operator results in a reflection with respect to the initial equiprobable superposition of all the legtimate states. Therefore, after a single application of Grover's operator, the resultant quantum state has been rotated clockwise by an angle of 2θ, where θ is described in (4). This phenomenon takes place each time Grover's operator is employed, even if the vector that described the quantum system with respect to the basis that comprises of $|s\rangle$ and $|ns\rangle$ moves to the second, third or fourth quadrant. The highest probability of observing a quantum state that is a solution occurs when the quantum system's vector has an angle of 90° with respect to the vector that consists of the quantum states that are not solutions $|ns\rangle$. The probability of obtaining a quantum state that constitutes a solution when we measure the final quantum state after L applications of Grover's operator was shown to be

$$P_{success} = \sin^2\left[(2L+1)\,\theta\right]. \tag{5}$$

Applying Grover's operator L_{opt} consecutive times results in a success probability close to unity, for high values of N, because we have

$$\begin{aligned} P_{success} &= \sin^2\left[(2L_{opt}+1)\,\theta\right] \\ &= \sin^2\left[\left(2\left\lfloor\frac{\pi}{4}\sqrt{\frac{N}{S}}\right\rfloor+1\right)\arcsin\left(\sqrt{\frac{S}{N}}\right)\right] \\ &> 0.99, \text{ when } S=1 \text{ and } n=\log_2(N)\geq 5. \end{aligned} \tag{6}$$

5 Depolarizing Channel Before the Oracle: An Example

Let us now investigate step by step in greater depth the deleterious effects of quantum perturbations by following an example search of an $N=8$-element database having $S=1$ solution. Without any loss of generality, let us assume that the known solution x_s is constituted by the state $|x_s\rangle = |1\rangle = |001\rangle$. The optimal number of Grover iterations would be $L_{opt}=2$. The

initial equiprobable superposition of all the $N = 8$ states is illustrated in Supplementary Fig. 2 as

$$\begin{aligned} |q\rangle &= \frac{1}{\sqrt{N}} (|000\rangle + |001\rangle + |010\rangle + |011\rangle \\ &\quad + |100\rangle + |101\rangle + |110\rangle + |111\rangle) \\ &= 0.354 \cdot (|0\rangle + |1\rangle + |2\rangle + |3\rangle + |4\rangle + |5\rangle + |6\rangle + |7\rangle) \,. \end{aligned} \tag{7}$$

Let us also assume that due to the realistic depolarizing perturbations of the environment, a phase flip error occurs at the third qubit, flipping the sign of all the specific quantum states, for which the third qubit is equal to $|1\rangle$, as encapsulated in

$$\begin{aligned} |q_1\rangle &= (I \otimes I \otimes Z)\,|q\rangle \\ &= 0.354 \cdot (|0\rangle|0\rangle(Z|0\rangle) + |0\rangle|0\rangle(Z|1\rangle) \\ &\quad + |0\rangle|1\rangle(Z|0\rangle) + |0\rangle|1\rangle(Z|1\rangle) \\ &\quad + |1\rangle|0\rangle(Z|0\rangle) + |1\rangle|0\rangle(Z|1\rangle) \\ &\quad + |1\rangle|1\rangle(Z|0\rangle) + |1\rangle|1\rangle(Z|1\rangle)) \\ &= 0.354 \cdot (|0\rangle|0\rangle|0\rangle + |0\rangle|0\rangle(-|1\rangle) \\ &\quad + |0\rangle|1\rangle|0\rangle + |0\rangle|1\rangle(-|1\rangle) \\ &\quad + |1\rangle|0\rangle|0\rangle + |1\rangle|0\rangle(-|1\rangle) \\ &\quad + |1\rangle|1\rangle|0\rangle + |1\rangle|1\rangle(-|1\rangle)) \\ &= 0.354 \cdot (|000\rangle - |001\rangle + |010\rangle - |011\rangle \\ &\quad + |100\rangle - |101\rangle + |110\rangle - |111\rangle) \\ &= 0.354 \cdot (|0\rangle - |1\rangle + |2\rangle - |3\rangle + |4\rangle - |5\rangle + |6\rangle - |7\rangle). \end{aligned} \tag{8}$$

In other words, all the odd-valued quantum states are tentatively marked as a solution to the search problem, due to this phase flip error, as encapsulated in $|q_1\rangle$ of Supplementary Fig. 2 and (8). Since the operation of the Oracle gate is assumed to be ideal, the specific quantum state of $|1\rangle = |001\rangle$, which is the unique correct solution of the search problem considered will be "dutifully" marked by having its sign flipped, resulting in $|q_2\rangle$ in Supplementary Fig. 2, as described by

$$\begin{aligned} |q_2\rangle &= O|q_1\rangle \\ &= 0.354 \cdot (|0\rangle - (-|1\rangle) + |2\rangle - |3\rangle \\ &\quad + |4\rangle - |5\rangle + |6\rangle - |7\rangle) \\ &= 0.354 \cdot (|0\rangle + |1\rangle + |2\rangle - |3\rangle + |4\rangle - |5\rangle + |6\rangle - |7\rangle). \end{aligned} \tag{9}$$

Essentially, this may be viewed as though the flawless Oracle, which is unaware of the perturbation has "unmarked" the correct solution, leaving only the quantum states $|3\rangle$, $|5\rangle$ and $|7\rangle$ acting as the potential solution states. Then, the diffusion operator is applied to $|q_2\rangle$, yielding the quantum state $|q_3\rangle$ as its output in Supplementary Fig. 2. The operation of the diffusion operator may be algebraically described by firstly calculating the average amplitude of the states superimposed in $|q_2\rangle$ of (9), which is equal to

$$\mu_{q_2} = \frac{5 \cdot 0.354 - 3 \cdot 0.354}{8} = 0.089. \tag{10}$$

Since the diffusion operator may be considered as a reflection of a state's amplitude with respect to the average amplitudes of the superimposed states, $|q_3\rangle$ of Supplementary Fig. 2 may be described as

$$\begin{aligned} |q_3\rangle &= (2\cdot\mu_{q_2} - 0.354)\cdot(|0\rangle + |1\rangle + |2\rangle + |4\rangle + |6\rangle) \\ &\quad + (2\cdot\mu_{q_2} + 0.354)\cdot(|3\rangle + |5\rangle + |7\rangle) \\ &= -0.177|0\rangle - 0.177|1\rangle - 0.177|2\rangle + 0.531|3\rangle \\ &\quad - 0.177|4\rangle + 0.531|5\rangle - 0.177|6\rangle + 0.531|7\rangle. \end{aligned} \tag{11}$$

Based on $|q_3\rangle$, there is a probability of

$$P_{|3\rangle,|5\rangle,|7\rangle} = (0.531)^2 + (0.531)^2 + (0.531)^2 = 0.846, \tag{12}$$

or 84.6% of observing one of the quantum states $|3\rangle$, $|5\rangle$ or $|7\rangle$, while only a probability of

$$P_{|1\rangle} = (-0.177)^2 = 0.0313, \tag{13}$$

or 3.13% of observing the actual solution state $|1\rangle$.

During the second application of Grover's operator in Supplementary Fig. 2, let us assume that a bit flip affects the first of the three information qubits. This may be translated into the first $N/2 = 4$ quantum states and the last $N/2 = 4$ quantum states swapping their amplitudes, as illustrated in $|q_4\rangle$ in Supplementary Fig. 2, where, for example, $|1\rangle$ and $|5\rangle$ have exchanged their amplitudes. Please note that in $|q_4\rangle$, the states $|1\rangle$, $|3\rangle$ and $|7\rangle$ are as if they had been marked to be solutions during the first Grover iteration, as encapsulated in

$$\begin{aligned} |q_4\rangle &= (X\otimes I\otimes I)|q_3\rangle \\ &= -0.177(X|0\rangle)|0\rangle|0\rangle - 0.177(X|0\rangle)|0\rangle|1\rangle \\ &\quad - 0.177(X|0\rangle)|1\rangle|0\rangle + 0.531(X|0\rangle)|1\rangle|1\rangle \\ &\quad - 0.177(X|1\rangle)|0\rangle|0\rangle + 0.531(X|1\rangle)|0\rangle|1\rangle \\ &\quad - 0.177(X|1\rangle)|1\rangle|0\rangle + 0.531(X|1\rangle)|1\rangle|1\rangle \\ &= -0.177|1\rangle|0\rangle|0\rangle - 0.177|1\rangle|0\rangle|1\rangle \\ &\quad - 0.177|1\rangle|1\rangle|0\rangle + 0.531|1\rangle|1\rangle|1\rangle \\ &\quad - 0.177|0\rangle|0\rangle|0\rangle + 0.531|0\rangle|0\rangle|1\rangle \\ &\quad - 0.177|0\rangle|1\rangle|0\rangle + 0.531|0\rangle|1\rangle|1\rangle \\ &= -0.177|4\rangle - 0.177|5\rangle - 0.177|6\rangle + 0.531|7\rangle \\ &\quad - 0.177|0\rangle + 0.531|1\rangle - 0.177|2\rangle + 0.531|3\rangle \\ &= -0.177|0\rangle + 0.531|1\rangle - 0.177|2\rangle + 0.531|3\rangle \\ &\quad - 0.177|4\rangle - 0.177|5\rangle - 0.177|6\rangle + 0.531|7\rangle. \end{aligned} \tag{14}$$

The application of the Oracle during the second Grover iteration will result in flipping the sign of the $|1\rangle$ state, as indicated by

$|q_5\rangle$ in Supplementary Fig. 2 and

$$\begin{aligned}|q_5\rangle &= O|q_4\rangle \\ &= -0.177|0\rangle + 0.531(-|1\rangle) - 0.177|2\rangle + 0.531|3\rangle \\ &\quad - 0.177|4\rangle - 0.177|5\rangle - 0.177|6\rangle + 0.531|7\rangle. \\ &= -0.177|0\rangle - 0.531|1\rangle - 0.177|2\rangle + 0.531|3\rangle \\ &\quad - 0.177|4\rangle - 0.177|5\rangle - 0.177|6\rangle + 0.531|7\rangle.\end{aligned} \tag{15}$$

This is deemed to alter the operation of Grover's QSA, since a third amplitude "level" is expected to appear after the diffusion operator. The reason behind it is that the diffusion operator reflects the amplitudes of all quantum states with respect to their collective average, which may be written for $|q_5\rangle$ as

$$\mu_{q_5} = \frac{-5 \cdot 0.177 + 2 \cdot 0.531 - 0.531}{8} = -0.044. \tag{16}$$

Therefore, after the second diffusion operator, the resultant superposition of states $|q_6\rangle$ illustrated in Supplementary Fig. 2 is described by

$$\begin{aligned}|q_6\rangle &= (2 \cdot \mu_{q_5} + 0.177) \cdot (|0\rangle + |2\rangle + |4\rangle + |5\rangle + |6\rangle) \\ &\quad + (2 \cdot \mu_{q_5} - 0.531) \cdot (|3\rangle + |7\rangle) \\ &\quad + (2 \cdot \mu_{q_5} + 0.531) \cdot |1\rangle \\ &= 0.089|0\rangle + 0.443|1\rangle + 0.089|2\rangle - 0.619|3\rangle \\ &\quad + 0.089|4\rangle + 0.089|5\rangle + 0.089|6\rangle - 0.619|7\rangle.\end{aligned} \tag{17}$$

In conclusion, in the example described in Supplementary Fig. 2, there is a probability of

$$P_{|1\rangle} = (0.443)^2 = 0.196, \tag{18}$$

or 19.6% of success according to (17), which corresponds to the scenario, where $|1\rangle$ is obtained after measuring $|q_6\rangle$.

6 Implementation of the Encoder & Decoder: An Example

6.1 Steane Code

The Steane code is a $[7,1]$ stabilizer code, which is capable of correcting a single error (bit-flip or phase-flip or both). It is derived from the dual-containing classical $(7,4)$ Hamming code $\mathscr{C}$, whose Parity Check Matrix (PCM) is

$$\mathbf{H} = \begin{pmatrix} 1 & 1 & 0 & 1 & 1 & 0 & 0 \\ 1 & 0 & 1 & 1 & 0 & 1 & 0 \\ 0 & 1 & 1 & 1 & 0 & 0 & 1 \end{pmatrix}. \tag{19}$$

The corresponding encoding circuit is given in Supplementary Fig. 3, which maps the states $|0\rangle$ and $|1\rangle$ of a logical qubit $|q\rangle$ onto the unique cosets of the dual code $\mathscr{C}^\perp$ in the code space of $\mathscr{C}$, where $\mathscr{C}^\perp$ is generated using the PCM of (19). More explicitly, the state $|0\rangle$ is mapped onto a uniform superposition of the codewords of the dual code $\mathscr{C}^\perp$ with the aid of six

auxiliary qubits, which may be mathematically formulated as

$$
\begin{aligned}
|0\rangle \rightarrow |\overline{0}\rangle &\equiv \frac{1}{\sqrt{8}} \sum_{\overline{c}\in\mathscr{C}^{\perp}} |\overline{c}\rangle \\
&= \frac{1}{\sqrt{8}} \sum_{\overline{q}^{(1)},\overline{q}^{(2)},\overline{q}^{(3)}} \Big(|\overline{q}^{(1)} \oplus \overline{q}^{(2)}\rangle |\overline{q}^{(1)} \oplus \overline{q}^{(3)}\rangle |\overline{q}^{(2)} \oplus \overline{q}^{(3)}\rangle \\
&\qquad \otimes |\overline{q}^{(1)} \oplus \overline{q}^{(2)} \oplus \overline{q}^{(3)}\rangle |\overline{q}^{(1)}\rangle |\overline{q}^{(2)}\rangle |\overline{q}^{(3)}\rangle \Big) \\
&= \frac{1}{\sqrt{8}} (|0000000\rangle + |0111001\rangle + |1011010\rangle + |1100011\rangle \\
&\quad + |1101100\rangle + |1010101\rangle + |0110110\rangle + |0001111\rangle),
\end{aligned} \tag{20}
$$

while the state $|1\rangle$ is encoded into a superposition of the codewords of a coset of $\mathscr{C}^{\perp}$ in $\mathscr{C}$, so that we have

$$
\begin{aligned}
|1\rangle \rightarrow |\overline{1}\rangle &\equiv \frac{1}{\sqrt{8}} \sum_{\overline{c}\in\mathscr{C}^{\perp}} |1110000 \oplus \overline{c}\rangle \\
&= \frac{1}{\sqrt{8}} (|1110000\rangle + |1001001\rangle + |0101010\rangle + |0010011\rangle \\
&\quad + |0011100\rangle + |0100101\rangle + |1000110\rangle + |1111111\rangle).
\end{aligned} \tag{21}
$$

The superimposed states of (20) and (21) together constitute the code space of the classical Hamming code. The stabilizer generators associated with the encoding states of (20) and (21) are listed in Supplementary Table 2. It may be observed in Supplementary Table 2 that in the first three rows, Z stabilizers are placed at the locations where $\mathbf{H}$ of (19) has a value of 1, while all other indices corresponding to a value of 0 in (19) are marked with an identity operator I. Similarly, an X operator is placed in rows 4, 5 and 6 at the locations of 1 in H of (19). Furthermore, Z stabilizers are used for detecting bit flips, while the X stabilizers are used for identifying phase flips. At the decoder, each stabilizer generator is implemented using an additional auxiliary qubit, as demonstrated in Supplementary Fig. 4, where the stabilizer g_1 of Supplementary Table 2 is used as an example.

Let us now investigate the role of the Steane code in counteracting decoherence by following the example of Supplementary Fig. 2. The initial equiprobable superposition of all the $N = 8$ states encapsulated in (7) may also be expressed as

$$
|q\rangle = |+\rangle \otimes |+\rangle \otimes |+\rangle, \tag{22}
$$

where $|+\rangle = H|0\rangle = \frac{1}{\sqrt{2}}(|0\rangle + |1\rangle)$ and $\otimes$ denotes the tensor product. Each of the three qubits of (22) is encoded using the Steane code, resulting in a 21-qubit superimposed state as shown at the encoder output of Supplementary Fig. 3:

$$
|\overline{q}\rangle = |\overline{+}\rangle \otimes |\overline{+}\rangle \otimes |\overline{+}\rangle, \tag{23}
$$

where the encoded qubits $|\overline{+}\rangle = \frac{1}{\sqrt{2}}(|\overline{0}\rangle + |\overline{1}\rangle)$, while $|\overline{0}\rangle$ and $|\overline{1}\rangle$ are the 7-qubit encoded states of (20) and (21). More explicitly,

we have

$$|\overline{+}\rangle \equiv \frac{1}{\sqrt{16}}(|0000000\rangle + |0111001\rangle + |1011010\rangle \\ + |1100011\rangle + |1101100\rangle + |1010101\rangle + |0110110\rangle \\ + |0001111\rangle + |1110000\rangle + |1001001\rangle + |0101010\rangle \\ + |0010011\rangle + |0011100\rangle + |0100101\rangle + |1000110\rangle \\ + |1111111\rangle). \tag{24}$$

Recall from Supplementary Fig. 2 that a phase flip error occurs at the third logical qubit under realistic depolarizing perturbations. Since each logical qubit is encoded into 7 qubits, the third logical qubit of $|q\rangle$ is the fifteenth qubit of $|\overline{q}\rangle$. Consequently, the sign of all states in (23), for which the fifteenth qubit is equal to $|1\rangle$, is flipped, resulting in

$$\begin{aligned}|\overline{q}_1\rangle &= (IIIIII \otimes IIIIII \otimes ZIIIII)\,|\overline{q}\rangle \\ &= (IIIIII \otimes IIIIII \otimes ZIIIII)\,(|\overline{+}\rangle \otimes |\overline{+}\rangle \otimes |\overline{+}\rangle) \\ &= |\overline{+}\rangle \otimes |\overline{+}\rangle \otimes \frac{1}{\sqrt{16}}(|0000000\rangle + |0111001\rangle - |1011010\rangle \\ &- |1100011\rangle - |1101100\rangle - |1010101\rangle + |0110110\rangle \\ &+ |0001111\rangle - |1110000\rangle - |1001001\rangle + |0101010\rangle \\ &+ |0010011\rangle + |0011100\rangle + |0100101\rangle - |1000110\rangle \\ &- |1111111\rangle).\end{aligned} \tag{25}$$

The perturbed states of (25) are decoded before being fed to the Oracle. Recall that decoding is a 3-step process, which proceeds as follows:

1. **Syndrome Processing:** The perturbed encoded qubits $|\overline{q}_1\rangle$ of (25) consist of three blocks of 7 qubits, each encoded independently using the Steane code. For the sake of computing the syndromes, the stabilizer generators of Supplementary Table 2 are applied individually to each block using auxiliary qubits, as demonstrated in Supplementary Fig. 4. Let us now take a closer look at the operations of Supplementary Fig. 4, assuming that the generator g_1 is applied to the first block of (25), i.e. $|\overline{+}\rangle$. An auxiliary qubit $|0\rangle$ is used in conjunction with the received codeword $|+\rangle$ as

$$\begin{aligned}|t_1\rangle &= H|0\rangle \otimes |\overline{+}\rangle \\ &= \frac{1}{\sqrt{2}}(|0\rangle + |1\rangle) \otimes |\overline{+}\rangle \\ &= \frac{1}{\sqrt{2}}(|0\rangle \otimes |\overline{+}\rangle + |1\rangle \otimes |\overline{+}\rangle)\end{aligned} \tag{26}$$

A series of controlled-Z gates are then applied to the first, second, fourth and fifth qubits of the received codeword, which are controlled by the auxiliary qubit. In other words, a Z gate is applied to the first, second, fourth and fifth qubits of the received codeword, only when the auxiliary control qubit is in the $|1\rangle$ state, as shown below:

$$|t_2\rangle = \frac{1}{\sqrt{2}}(|0\rangle \otimes |\overline{+}\rangle + (|1\rangle \otimes (IZZIZZII)|\overline{+}\rangle)). \tag{27}$$

Consequently, the sign of all states of (26), for which odd number of 1's are present at the first, second, fourth and fifth index of the received codeword, is flipped. Since the first block is a valid codeword, none of the superimposed states of

(26) have an odd number of 1's at the first, second, fourth and fifth index of the received codeword. This results in

$$|t_2\rangle = \frac{1}{\sqrt{2}}(|0\rangle \otimes |\mp\rangle + |1\rangle \otimes |\mp\rangle) \\ = \frac{1}{\sqrt{2}}(|0\rangle + |1\rangle) \otimes |\mp\rangle. \tag{28}$$

Applying the Hadamard gate again to the auxiliary qubit, reverts its value to $|0\rangle$, as in

$$|t_3\rangle = H\frac{1}{\sqrt{2}}(|0\rangle + |1\rangle) \otimes |\mp\rangle = |0\rangle \otimes |\mp\rangle. \tag{29}$$

However, if there had been a single bit error on either the first or the second or the fourth or the fifth index of the codeword, then the application of g_1 to the received codeword would have yielded

$$|t_2\rangle = \frac{1}{\sqrt{2}}|0\rangle \otimes |\mp\rangle - |1\rangle \otimes |\mp\rangle \\ = \frac{1}{\sqrt{2}}(|0\rangle - |1\rangle) \otimes |\mp\rangle, \tag{30}$$

rather than (28). In the context of (30), the auxiliary qubit would assume the state $|1\rangle$ following the application of the Hadamard gate, as stated in

$$|t_3\rangle = H\frac{1}{\sqrt{2}}(|0\rangle - |1\rangle) \otimes |\mp\rangle = |1\rangle \otimes |\mp\rangle. \tag{31}$$

The syndrome sequence corresponding to the stabilizer generators of Supplementary Table 2 may be used in conjunction with the look-up table of Supplementary Table 3 for identifying the single qubit errors in each of the three 7-qubit blocks of (25). Since in our example the first two blocks of $|\overline{q}_1\rangle$ are error-free, all the stabilizer generators of Supplementary Table 2 give a zero-valued syndrome, when applied to these two blocks. However, a phase flip error occurred on the first qubit of the third block in our example. Consequently, the stabilizer generators g_4 and g_5 yield a syndrome value of 1 for the third block of $|\overline{q}_1\rangle$, while all the remaining generators yield a zero-valued syndrome. Hence, based on the syndrome value, it may be identified that a phase flip error occurred on the first qubit of the third block.

2. **Error Recovery:** The impact of phase error is reversed by applying a Pauli-Z gate to the first qubit of the third block, so that we have

$$|\tilde{\overline{q}}\rangle = (IIIIIII \otimes IIIIIII \otimes ZIIIIII)\,|\overline{q}_1\rangle = |\overline{q}\rangle. \tag{32}$$

3. **Inverse Encoding:** The recovered encoded qubits $|\tilde{\overline{q}}\rangle$ are passed through the circuit of Supplementary Fig. 3, operating from right to left, in blocks of 7-qubits, yielding the original logical qubits $|q\rangle$.

Hence, with the aid of the Steane code, the deleterious effect of phase errors, which was demonstrated in Supplementary Fig. 2, may be counteracted. Similarly, the bit flip error encountered during the second iteration of Grover's operator in Supplementary Fig. 2 may be counteracted by encoding and decoding the qubits $|q_4\rangle$ before feeding them to the oracle.

6.2 Quantum BCH Code

Analogously to the Steane code, according to Supplementary Fig. 5, the stabilizers of QBCH$[15,7]$ are constructed using the PCM of

$$\begin{aligned}\mathbf{H} &= [\mathbf{I}_{\mathrm{m}}|\mathbf{P}] \\ &= \left(\begin{array}{cccc|ccccccccccc} 1 & 0 & 0 & 0 & 1 & 1 & 1 & 1 & 0 & 1 & 0 & 1 & 1 & 0 & 0 \\ 0 & 1 & 0 & 0 & 0 & 1 & 1 & 1 & 1 & 0 & 1 & 0 & 1 & 1 & 0 \\ 0 & 0 & 1 & 0 & 0 & 0 & 1 & 1 & 1 & 1 & 0 & 1 & 0 & 1 & 1 \\ 0 & 0 & 0 & 1 & 1 & 1 & 1 & 0 & 1 & 0 & 1 & 1 & 0 & 0 & 1 \end{array}\right).\end{aligned} \tag{33}$$

by replacing 1's with Z (or X), while 0's are replaced with I. The resultant stabilizer generators are listed in Supplementary Table 4.

7 Oracle in Grover's algorithm: An Example

Assuming that we have a database of size $N = 8$ and that only $Z = 3$ qubits are used for representing $f(x)$ and δ, let us consider

$$\delta = 010. \tag{34}$$

Let us also assume that the function's values for the legitimate entries of $f(\cdot)$ are the ones stated in Supplementary Table 1. Therefore, after the application of the first Z number of CNOT gates in the Oracle, the entangled superimposed states of the system may be described as

$$\begin{aligned}|w_2\rangle &= \frac{1}{\sqrt{N}}\left(\sum_{x=0}^{N-1}|x\rangle CNOT\left(|f(x)\rangle|\delta\rangle\right)\right)|-\rangle \\ &= \frac{1}{\sqrt{N}}\left(\sum_{x=0}^{N-1}|x\rangle|f(x)\rangle|f(x)\oplus\delta\rangle\right)|-\rangle \\ &= \frac{1}{\sqrt{8}}(|000\rangle|011\rangle|0\oplus 0\rangle|1\oplus 1\rangle|1\oplus 0\rangle \\ &\quad +|001\rangle|010\rangle|0\oplus 0\rangle|1\oplus 1\rangle|0\oplus 0\rangle \\ &\quad +|010\rangle|111\rangle|1\oplus 0\rangle|1\oplus 1\rangle|1\oplus 0\rangle \\ &\quad +|011\rangle|000\rangle|0\oplus 0\rangle|0\oplus 1\rangle|0\oplus 0\rangle \\ &\quad +|100\rangle|111\rangle|1\oplus 0\rangle|1\oplus 1\rangle|1\oplus 0\rangle \\ &\quad +|101\rangle|110\rangle|1\oplus 0\rangle|1\oplus 1\rangle|0\oplus 0\rangle \\ &\quad +|110\rangle|011\rangle|0\oplus 0\rangle|1\oplus 1\rangle|1\oplus 0\rangle \\ &\quad +|111\rangle|001\rangle|0\oplus 0\rangle|0\oplus 1\rangle|1\oplus 0\rangle)|-\rangle \\ &= \frac{1}{\sqrt{8}}(|000\rangle|011\rangle|001\rangle+|001\rangle|010\rangle|000\rangle \\ &\quad +|010\rangle|111\rangle|101\rangle+|011\rangle|000\rangle|010\rangle \\ &\quad +|100\rangle|111\rangle|101\rangle+|101\rangle|110\rangle|100\rangle \\ &\quad +|110\rangle|011\rangle|001\rangle+|111\rangle|001\rangle|011\rangle)|-\rangle,\end{aligned} \tag{35}$$

verifying that the solution state is the one entangled with the reference register being in the all-zero state, which in our scenario is $|x\rangle = |001\rangle$. The Pauli-X operators, which follow the CNOT gates will apply an unconditional bit flip to the qubits of the reference register, resulting in

$$\begin{aligned}|w_3\rangle = \frac{1}{\sqrt{8}}(&|000\rangle|011\rangle|110\rangle + |001\rangle|010\rangle|111\rangle \\ &+ |010\rangle|111\rangle|010\rangle + |011\rangle|000\rangle|101\rangle \\ &+ |100\rangle|111\rangle|010\rangle + |101\rangle|110\rangle|011\rangle \\ &+ |110\rangle|011\rangle|110\rangle + |111\rangle|001\rangle|100\rangle)|-\rangle.\end{aligned} \tag{36}$$

Therefore, by applying a multi-controlled NOT gate, where all control qubits have to be in the $|1\rangle$ in order for the value qubit to perform a bit flip, the sign of the auxiliary qubit $|-\rangle$ will be flipped only when entangled with a reference register in the all-one state $|1\rangle^{\otimes Z} = |111\rangle$, as stated in

$$\begin{aligned}|w_4\rangle = \frac{1}{\sqrt{8}}(&|000\rangle|011\rangle|110\rangle|-\rangle + |001\rangle|010\rangle|111\rangle(-|-\rangle) \\ &+ |010\rangle|111\rangle|010\rangle|-\rangle + |011\rangle|000\rangle|101\rangle|-\rangle \\ &+ |100\rangle|111\rangle|010\rangle|-\rangle + |101\rangle|110\rangle|011\rangle|-\rangle \\ &+ |110\rangle|011\rangle|110\rangle|-\rangle + |111\rangle|001\rangle|100\rangle|-\rangle). \\ = \frac{1}{\sqrt{8}}(&|000\rangle|011\rangle|110\rangle - |001\rangle|010\rangle|111\rangle \\ &+ |010\rangle|111\rangle|010\rangle + |011\rangle|000\rangle|101\rangle \\ &+ |100\rangle|111\rangle|010\rangle + |101\rangle|110\rangle|011\rangle \\ &+ |110\rangle|011\rangle|110\rangle + |111\rangle|001\rangle|100\rangle)|-\rangle.\end{aligned} \tag{37}$$

References

1. Botsinis, P., Ng, S. X. & Hanzo, L. Quantum search algorithms, quantum wireless, and a low-complexity maximum likelihood iterative quantum multi-user detector design. *IEEE Access* **1**, 94–122 (2013).
2. Botsinis, P., Ng, S. X. & Hanzo, L. Fixed-complexity quantum-assisted multi-user detection for CDMA and SDMA. *IEEE Transactions on Communications* **62**, 990–1000 (2014).
3. Botsinis, P., Alanis, D., Ng, S. X. & Hanzo, L. Low-complexity soft-output quantum-assisted multiuser detection for direct-sequence spreading and slow subcarrier-hopping aided SDMA-OFDM systems. *IEEE Access* **2**, 451–472 (2014).
4. Botsinis, P., Alanis, D., Babar, Z., Ng, S. & Hanzo, L. Iterative quantum-assisted multi-user detection for multi-carrier interleave division multiple access systems. *IEEE Transactions on Communications* **63**, 3713–3727 (2015).
5. Botsinis, P., Alanis, D., Babar, Z., Ng, S. X. & Hanzo, L. Noncoherent quantum multiple symbol differential detection for wireless systems. *IEEE Access* **3**, 569–598 (2015).
6. Durr, C. & Høyer, P. A quantum algorithm for finding the minimum. *Quantum Physics* 9607014 (1996).
7. Gan, G., Ma, C. & Wu, J. *Data clustering: theory, algorithms, and applications*, vol. 20 (Siam, 2007).
8. Yang, D., Yang, L. L. & Hanzo, L. Performance of SDMA systems using transmitter preprocessing based on noisy feedback of vector-quantized channel impulse responses. *IEEE Vehicular Technology Conference* 2119–2123 (2007).
9. Hastie, T. J., Tibshirani, R. J. & Friedman, J. H. *The Elements of Statistical Learning : Data Mining, Inference, and Prediction* (Springer, 2009).

10. Lu, J., Wang, G. & Moulin, P. Human identity and gender recognition from gait sequences with arbitrary walking directions. *IEEE Transactions on Information Forensics and Security* **9**, 51–61 (2014).

11. Matovski, D. S., Nixon, M. S., Mahmoodi, S. & Carter, J. N. The effect of time on gait recognition performance. *IEEE Transactions on Information Forensics and Security* **7**, 543–552 (2012).

12. Aïmeur, E., Brassard, G. & Gambs, S. Quantum speed-up for unsupervised learning. *Machine Learning* **90**, 261–287 (2012).

13. Wiebe, N., Kapoor, A. & Svore, K. Quantum algorithms for nearest-neighbor methods for supervised and unsupervised learning. *Quantum Information & Computation* **15**, 316–356 (2015).

14. Du, S., Yan, Y. & Ma, Y. Quantum-accelerated fractal image compression: an interdisciplinary approach. *IEEE Signal Processing Letters* **22**, 499–503 (2015).

15. Aquino, A., Gegundez-Arias, M. E. & Marin, D. Detecting the optic disc boundary in digital fundus images using morphological, edge detection, and feature extraction techniques. *IEEE Transactions on Medical Imaging* **29**, 1860–1869 (2010).

16. Lloyd, S., Garnerone, S. & Zanardi, P. Quantum algorithms for topological and geometric analysis of data. *Nature Communications* **7**, 10138 (2016).

17. Goldberg, D. E. *Genetic Algorithms in Search, Optimization and Machine Learning* (Addison-Wesley Professional, 1989).

18. Kennedy, J. & Eberhart, R. Particle swarm optimization. *IEEE International Conference on Neural Networks* **4**, 1942–1948 (1995).

19. Malossini, A., Blanzieri, E. & Calarco, T. Quantum genetic optimization. *IEEE Transactions on Evolutionary Computation* **12**, 231–241 (2008).

20. Jiang, M., Akhtman, J. & Hanzo, L. Iterative joint channel estimation and multi-user detection for multiple-antenna aided OFDM systems. *IEEE Transactions on Wireless Communications* **6**, 2904–2914 (2007).

21. Zhang, J., Chen, S., Mu, X. & Hanzo, L. Evolutionary-algorithm-assisted joint channel estimation and turbo multiuser detection/decoding for OFDM/SDMA. *IEEE Transactions on Vehicular Technology* **63**, 1204–1222 (2014).

22. Yao, W., Chen, S. & Hanzo, L. Generalized MBER-based vector precoding design for multiuser transmission. *IEEE Transactions on Vehicular Technology* **60**, 739–745 (2011).

23. Masouros, C., Sellathurai, M. & Ratnarajah, T. Vector perturbation based on symbol scaling for limited feedback MISO downlinks. *IEEE Transactions on Signal Processing* **62**, 562–571 (2014).

24. Yetgin, H., Cheung, K. T. K. & Hanzo, L. Multi-objective routing optimization using evolutionary algorithms. *IEEE Wireless Communications and Networking Conference* 3030–3034 (2012).

25. Alanis, D., Botsinis, P., Ng, S. X. & Hanzo, L. Quantum-assisted routing optimization for self-organizing networks. *IEEE Access* **2**, 614–632 (2014).

26. Alanis, D., Botsinis, P., Babar, Z., Ng, S. X. & Hanzo, L. Non-Dominated Quantum Iterative Routing Optimization for Wireless Multihop Networks. *IEEE Access* **3**, 1704–1728 (2015).
